# rt269L-Type hepatitis B virus (HBV) in genotype C infection leads to improved mitochondrial dynamics via the PERK–eIF2α–ATF4 axis in an HBx protein-dependent manner

**DOI:** 10.1186/s11658-023-00440-1

**Published:** 2023-03-30

**Authors:** Yu-Min Choi, Dong Hyun Kim, Junghwa Jang, Won Hyeok Choe, Bum-Joon Kim

**Affiliations:** 1 Department of Microbiology and Immunology, College of Medicine, Seoul National University, Seoul, 110-799 Republic of Korea; 2 Department of Internal Medicine, Konkuk University School of Medicine, Seoul, 05030 Republic of Korea; 3 Department of Biomedical Sciences, College of Medicine, Seoul National University, Seoul, 03080 Republic of Korea; 4 Liver Research Institute, College of Medicine, Seoul National University, Seoul, 03080 Korea; 5 Cancer Research Institute, College of Medicine, Seoul National University, Seoul, 03080 Korea; 6 Seoul National University Medical Research Center (SNUMRC), Seoul, 03080 Korea

**Keywords:** Mitochondrial functionality, ER stress, Autophagy, HBx stability, Deubiquitination

## Abstract

**Background:**

In our previous report, the rt269I type versus the rt269L type in genotype C2 infection led to poor clinical outcomes and enhanced mitochondrial stress in infected hepatocytes. Here, we sought to investigate differences between the rt269L and rt269I types in mitochondrial functionality in hepatitis B virus (HBV) genotype C2 infection, mainly focusing on endoplasmic reticulum (ER) stress-mediated autophagy induction as an upstream signal.

**Methods:**

Mitochondrial functionality, ER stress signaling, autophagy induction, and apoptotic cell death between rt269L-type and rt269I-type groups were investigated via in vitro and in vivo experiments. Serum samples were collected from 187 chronic hepatitis patients who visited Konkuk or Seoul National University Hospital.

**Results:**

Our data revealed that genotype C rt269L versus rt269I infection led to improved mitochondrial dynamics and enhanced autophagic flux, mainly due to the activation of the PERK–eIF2α–ATF4 axis. Furthermore, we demonstrated that the traits found in genotype C rt269L infection were mainly due to increased stability of the HBx protein after deubiquitination. In addition, clinical data using patient sera from two independent Korean cohorts showed that, compared with rt269I, rt269L in infection led to lower 8-OHdG levels, further supporting its improved mitochondrial quality control.

**Conclusion:**

Our data showed that, compared with the rt269I type, the rt269L type, which presented exclusively in HBV genotype C infection, leads to improved mitochondrial dynamics or bioenergetics, mainly due to autophagy induction via activation of the PERK–eIF2α–ATF4 axis in an HBx protein-dependent manner. This suggests that HBx stability and cellular quality control in the rt269L type predominating in genotype C endemic areas could at least partly contribute to some distinctive traits of genotype C infection, such as higher infectivity or longer duration of the hepatitis B e antigen (HBeAg) positive stage.

**Graphical Abstract:**

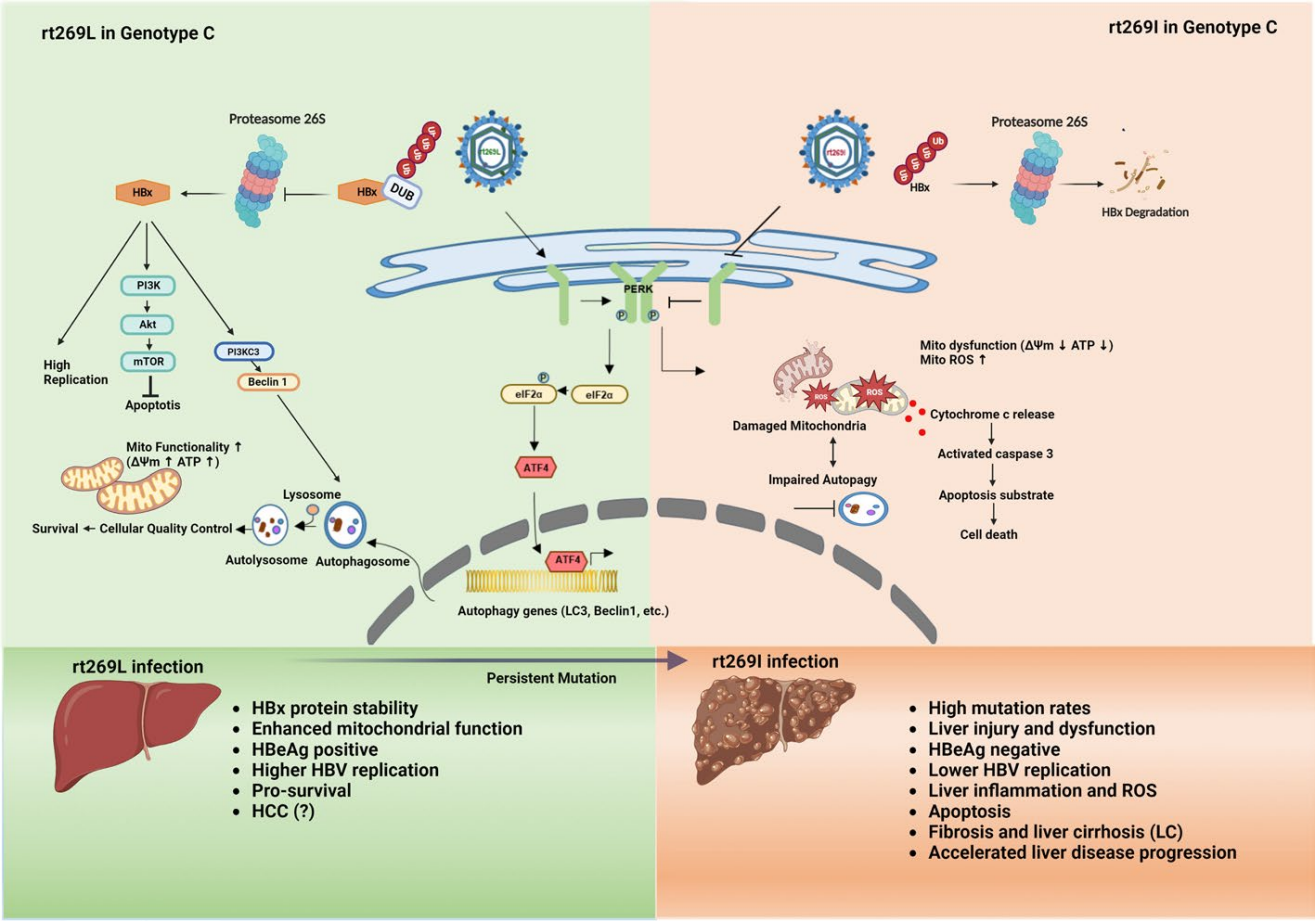

**Supplementary Information:**

The online version contains supplementary material available at 10.1186/s11658-023-00440-1.

## Background

Hepatitis B virus (HBV) infection is a major cause of chronic liver disease, including liver cirrhosis (LC), hepatic decompensation, and hepatocellular carcinoma (HCC) [1, 2]. Despite effective HBV vaccines and life cycle inhibitors, the annual number of HBV-related liver disease deaths is approximately 820,000 worldwide [3]. Based on the genetic divergence of the HBV genomic sequences, ten HBV genotypes (from A to J) and derived subtypes have been identified. The major genotypes diverge in terms of geographical distribution. Genotype A is most common in America, Africa, Europe and India. Genotypes B and C are highly prevalent in the Asia–Pacific region. Genotype D is widespread in Africa, Europe, India, and the Mediterranean region. Genotype E is limited to West and Central Africa, and Saudi Arabia. Genotype F is present in Central and South America, and Mexico. Genotype G has been found in the Americas, France, and Germany. Genotype H has been identified in Central America and Mexico. Genotype I is restricted to Laos and Vietnam. Genotype J is found in Japan [4, 5]. Among the 10 (A–J) HBV genotypes, infection with genotype C carries a greater risk for severe liver disease progression, with clinical features of prolonged active disease and delayed hepatitis B e antigen (HBeAg) seroconversion [6, 7]. Although clinical data and knowledge indicate poor prognosis for people with HBV genotype C infection, much remains unknown and requires clarification.

A substantial level of research has reported that there are clinical relationships between HBV infection and mitochondrial function. Since HBV does not have a metabolic system, it is crucial for HBV to utilize and control cell signal transduction for replication cycle success. Mitochondria are the most important intracellular organelles in cellular energy production and engage in key intracellular interactions with other organelles. Mitochondria can adapt to hostile cell environments by mitochondrial dynamics and mitophagy to maintain their homeostasis [8, 9]. Disruption of normal mitochondrial dynamics, mainly induced by pathological conditions, such as cancers and viral infection, can cause mitochondrial damage, leading to loss of its membrane potential and release of mitochondrial contents, including release of mitochondrial DNA (mtDNA) and/or ROS, into the blood, which ultimately cause proapoptotic or necrotic events [10, 11]. Mitochondrial damage and oxidative stress are prominent features of HBV infection. Furthermore, some preS or hepatitis B s antigen (HBsAg) variants causing endoplasmic reticulum (ER) stress-mediated liver disease progression have been reported to lead to mitochondrial dysfunction [12]

Recently, protein kinase R (PKR)-like ER kinase (PERK), an ER stress sensor involved in the unfolded protein response (UPR), has been reported to protect mitochondrial homeostasis during ER stress [13–15]. Activation of PERK–eIF2α–ATF4 can strengthen mitochondrial quality control by increasing mitochondrial biogenesis and mitophagy, resulting in renewal of the mitochondrial network [16].

There are two polymorphisms in HBV polymerase, rt269L and rt269I, in genotype C infection, distinct from other genotypes, of which most infection is due to a single rt269I type. Previously, we reported that the rt269I type leads to enhanced mitochondrial stress and was associated with a significantly greater extent with HBeAg-negative seroconversion and liver disease progression in genotype C infections than the rt269L type [17]. We also recently revealed that rt269I is more prone to mutation in the genotype C2 genome than rt269L, suggesting that the former could induce enhanced immune pressure during chronic infections [18]. Together, these results suggest that there may be definitely different capacities to maintain mitochondrial homeostasis between rt269L and rt269I types in genotype C infection. Elucidation of its underlying mechanism at the molecular level could provide a deeper understanding regarding distinct clinical and virological traits of HBV C infection. So, in the present study, we sought to investigate differences between the rt269L and rt269I types in mitochondrial functionality in HBV genotype C2 infection, mainly focusing on ER stress-mediated autophagy induction as its upstream signal.

## Methods

### Cells and reagents

Human hepatocellular carcinoma cells, HepG2 (#88065) and Huh7 (#60104) cells were purchased from the Korean Cell Line Bank (KCLB, Seoul, South Korea). HepG2 cells were maintained in Eagle’s minimum essential medium (MEM) containing 10% fetal bovine serum (FBS), 100 U/ml penicillin/streptomycin (PS), and 25 mM *N*-2-hydroxyethylpiperazine-*N*-2-ethanesulfonic acid (HEPES). Huh-7 cells were maintained in RPMI-1640 medium containing 10% FBS and 100 U/ml PS. HepG2-hNTCP-C4 cells were kindly gifted by Dr. Koichi Watashi from National Institute of Infectious Disease (Tokyo, Japan). HepG2-hNTCP-C4 cells were maintained in DMEM/F-12 supplemented with GlutaMAX, 10% FBS, 100 U/ml PS, 10 mM HEPES, 5 µg/ml insulin, and 500 µg/ml G418. HepaRG cells (HPR116) were purchased from Biopredic international (Saint-Gregoire, France) and were maintained in basal hepatic cell medium (MIL600C, Biopredic, Saint-Gregoire, France) with additives for maintenance/metabolism HepaRG medium (ADD620C, Biopredic, Saint-Gregoire, France).

### In vivo assay and hydrodynamic injection

C57BL/6 mice (7-week-old males) were hydrodynamically injected in the tail vein with 10 μg of pHBV-1.2x-rt269L [wild-type (WT), GenBank accession no. AY641558] or pHBV-1.2x-rt269I (generated by site-directed mutagenesis) plasmids carrying the full-length HBV genotype C genome in a volume of saline, which was equivalent to 10% of the mouse body weight. The total volume was delivered within 5–8 s. The mice were sacrificed 4 days after HBV-encoding DNA injection, and liver and serum were collected for analysis. All animal experiments were approved by the Institutional Animal Care and Use Committee (IACUC) of Seoul National University College of Medicine (SNU-210301-1-1).

### Patients, HBV DNA extraction, and PCR amplification of the polymerase RT region

For this study, serum samples were collected from 90 (KU) or 97 (SNU3) patients with chronic hepatitis B (CHB); all patients were diagnosed with chronic hepatitis B, and it was confirmed that no treatment such as nucleos(t)ide analogs (NAs) or interferon was initiated (Additional file 1: Table S1, Additional file 2: Table S2). These selection criteria include HBV s antigen (HBsAg) positive results for more than 6 months and detection of HBV DNA. Patients were excluded in the case of hepatitis C infection or coinfection of acquired immunodeficiency syndrome (AIDS), and autoimmune liver disease, alcohol or drug addiction. Serum was stored at −80 °C and used in this study. Viral DNA extracted from serum was stored at −20 °C and used for experiments. To determine the correlation between the polymorphism at the 269th amino acid and characteristics of disease, clinical factors and polymerase RT regions were assessed. This report was approved by the Institutional Review Board of Konkuk University Hospital (KUH-1010544) and Seoul National University Hospital (1012-131-346 and 1808-067-965). For this cohort study, HBV DNA was extracted from the serum of patients using a QIAamp DNA Blood Mini kit (QIAGEN, Hilden, Germany), and the sample was dissolved in Tris–EDTA buffer (10 mM Tris–HCl and 1 mM ethylenediaminetetraacetic acid, pH 8.0). First round of polymerase chain reaction (PCR) was performed using primers POL-RT1 and the amplicon was used as a template for the second round of PCR using primers POL-RT2 (Additional file 3: Table S3). The PCR products were subjected to direct sequencing analysis.

### Statistical analysis

The experimental data were analyzed with GraphPad Prism 9 (GraphPad Software, La Jolla, CA, USA). All experiments were independently repeated three times, and statistical analysis results are indicated in the figure legends. The *p* value indicating statistical significance was set at ^*^p < 0.05, ^**^p < 0.01, or ^***^p < 0.001.

A detailed description of the materials and methods is provided in Additional file 18.

## Results

### rt269L leads to improved mitochondrial maintenance with increased functional mitochondria and bioenergetics

**Fig. 1:**
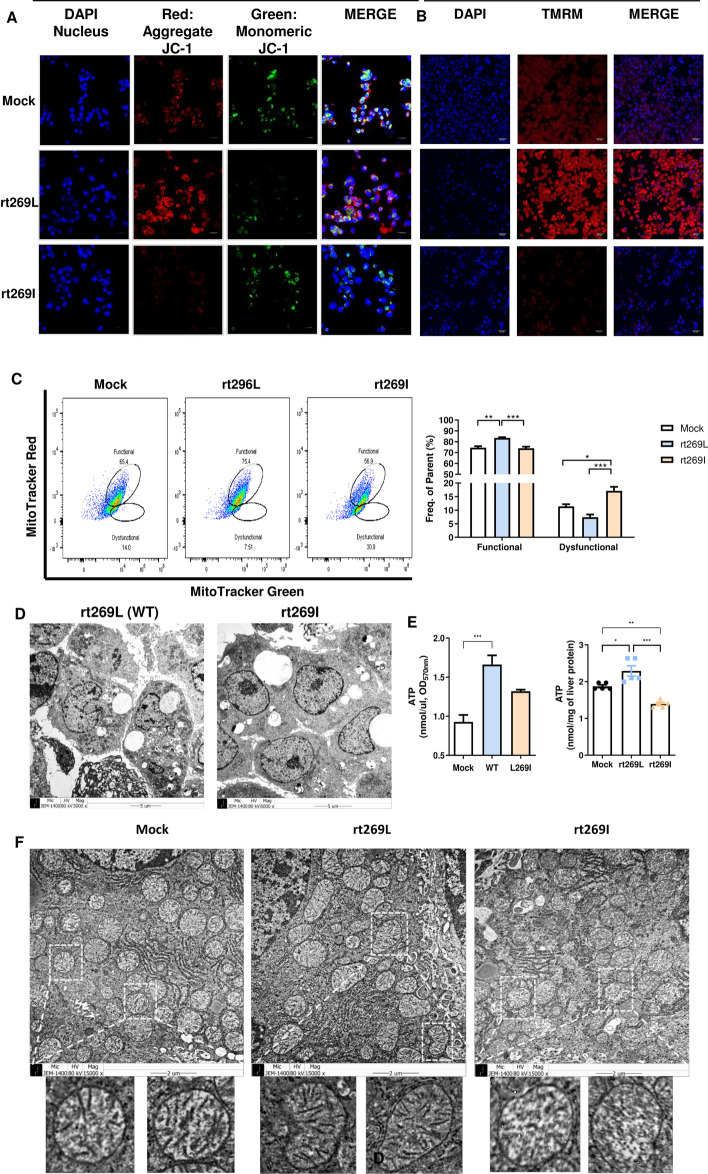
rt269L-infected cells showed improved mitochondrial maintenance with a high rate of functional mitochondria and biogenesis. **A, B** Confocal microscopy images showing HepG2 cells transfected with mock, rt269L, or rt269I HBV followed by JC-1 staining and TMRM staining indicating mitochondrial outer membrane permeabilization (MOMP) and ΔΨm. Nuclei were stained with DAPI (blue). Scale bar, 50 µm. **C** Evaluation of mitochondrial function by flow cytometry. Error shows the means ± stand error of the means (SEMs) *p < 0.05, **p < 0.01, ***p < 0.001. **D** High magnification of mitochondria in rt269L- and rt269I-infected HepG2-NTCP-C4 cells (scale bars, 5 or 1 µm). **E** Mitochondrial ATP generation in HepG2 cells transfected with mock, rt269L, or rt269I vector, or in liver tissues of mice hydrodynamically injected with mock, rt269L, or rt269I vector. **F** Mitochondrial cristae morphology and density in liver tissues of mice hydrodynamically injected with mock, rt269L, or rt269I HBV, assayed by transmission electron microscopy (TEM). Scale bar, 2 µm. The results were evaluated for statistical significance by one-way ANOVA with Tukey’s post hoc test. Differences were considered significant when *p < 0.05, **p < 0.01, and ***p < 0.001

Previously, we reported that the rt269I HBV variant versus rt269L led to liver disease progression, as indicated by enhanced mitochondrial stress and reactive oxygen species (ROS) production levels [17]. Therefore, in this study, we investigated mitochondrial features and their functionality in two types of HBV genotype C2 infection. First, the mitochondrial membrane potential (ΔΨm) was measured by JC-1 staining, a potential-sensitive fluorescent dye. As shown in Fig. 1A, rt269L infection led to greater emission of red fluorescence and lower emission of green florescence compared with rt269I. In addition, TMRM, another cell-permeant dye that accumulates in active mitochondria with intact membranes, was also more intense in the mitochondria of rt269L-infected cells than in rt269I-infected cells (Fig. 1B, Additional file 5: Fig. S1). Then, we analyzed the mitochondrial functionality in cells infected with each HBV type by flow cytometry based on mitochondrial mass (green fluorescence) and membrane potential (red fluorescence). Our data revealed that rt269L-infected cells contained a higher percentage of functional mitochondria (green^high^ and red^high^) and a lower percentage of dysfunctional mitochondria (green^high^ and red^low^) than rt269I-infected cells (Fig. 1C).

Next, intracellular mitochondrial biogenesis in HepG2-NTCP-C4 cells infected with rt269L or rt269I HBV virions was assessed by transmission electron microscopy (TEM). As shown in Fig. 1D, rt269L-infected cells contained increased numbers of mitochondria compared with rt269I-infected cells. mtDNA copy number is closely associated with cellular energy production [19], and PGC-1α is known to promote ATP production and energy homeostasis [20]. Our data indicated that rt269L induced increased mtDNA gene transcription, as well as increased PGC-1α protein expression (Additional file 6: Fig. S2). Consistently, rt269L infection enhanced ATP production in HepG2 cells and liver tissues of the model mice (Fig. 1E). Moreover, the mitochondrial cristae structures differed in mouse livers infected with rt269L or rt269I HBV. As shown in Fig. 1F, the liver tissue in mice injected with rt269L HBV contained mitochondria with normal and dense cristae structures. However, tissue exposed to rt269I HBV infection showed loss of normal cristae and many hollow areas, suggesting critically damaged inner mitochondrial matrices. Taken together, these results suggest that rt269L infection showed improved mitochondrial functionality with increased mitochondrial biogenesis and bioenergetics, while the rt269I variant led to impaired mitochondrial functionality.

### rt269L activates PERK–peIF2α–ATF4 signaling

**Fig. 2:**
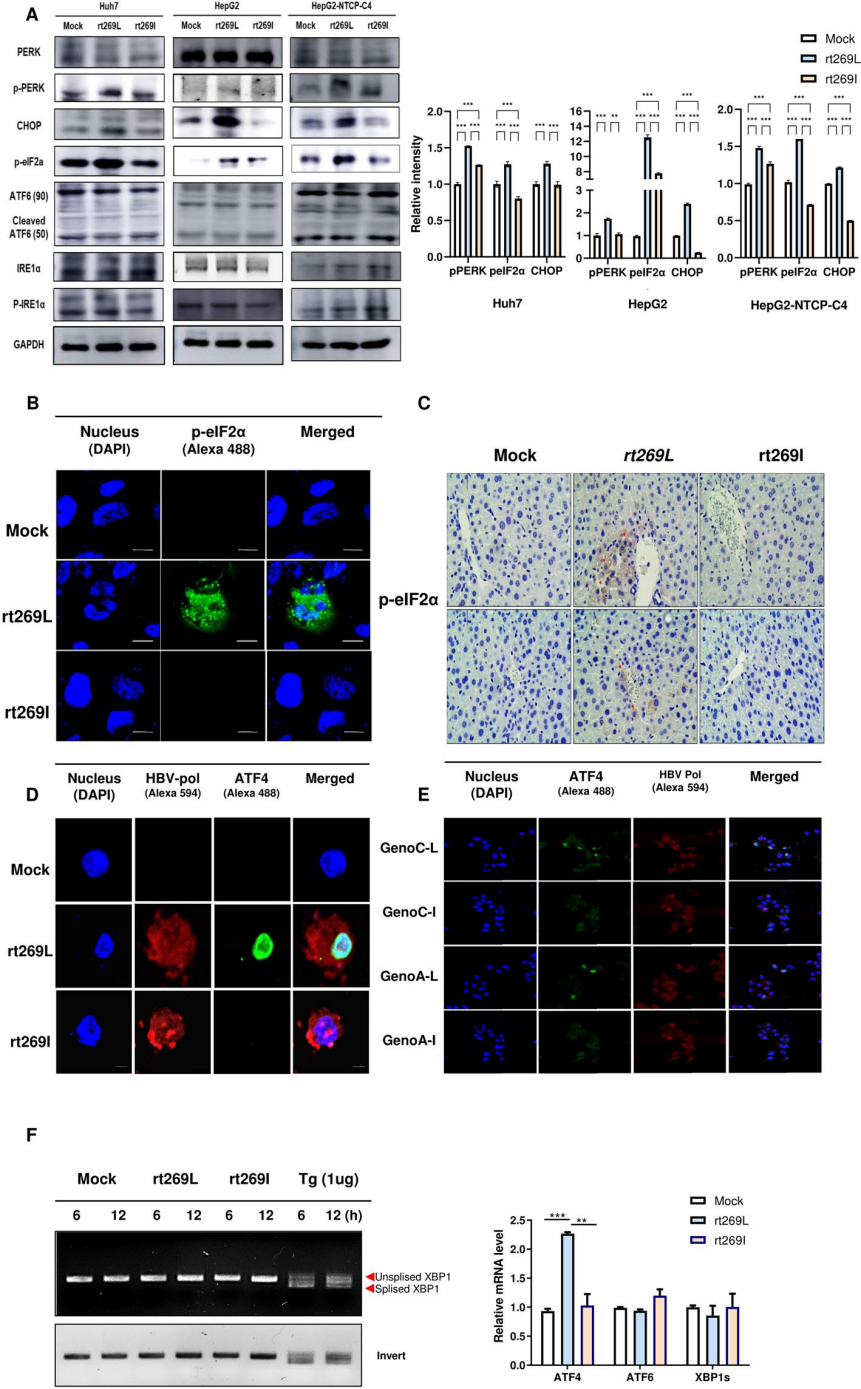
rt269L activates the PERK-mediated UPR pathway, but an impaired UPR is shown in rt269I-infected cells. **A** Markers of ER stress in Huh7, HepG2, and HepG2-NTCP-C4 cells, as measured by western blotting, after transfection or infection with mock, rt269L, or rt269I HBV. Some blots were cropped to improve the clarity and conciseness of the presentation. **B** Phosphorylation of eIF2α determined by immunofluorescence. HepG2 cells were transfected with a mock, rt269L, or rt269I vector and then immunostained with antibodies and DAPI and viewed by confocal microscopy. Scale bar, 10 µm. **C** Immunohistochemical analysis of p-eIF2α expression (red arrow) in paraffin-embedded liver tissues (magnification 100 ×, *n* = 5 per group). **D**, **E** ATF4 and HBV polymerase expression determined by immunofluorescence. **F** Left panel: agarose gel (3%) electrophoresis of XBP1 fragments (unspliced 295 bp, spliced 269 bp) from RNA isolation from HepG2 cells transfected with mock, rt269L, or rt269I HBV, or treated with thapsigargin (Tg, 1 µg/ml) for 6 and 12 h; right panel: RT–qPCR analysis of the transcription levels of UPR and ER stress-related genes 10 h posttransfection. Bar graph representing the relative expression levels (folds) of ATF4, ATF6, and sXBP1 calculated by the 2^−ΔΔCt^ method using β-actin as an endogenous control. **p < 0.01, ***p < 0.001

Mitochondrial functionality is closely related to cellular signals, including ER stress signals [21]. As we verified a significant difference in mitochondrial functionality between rt269L and rt269I HBV infection, we investigated whether the ER stress induction levels differed. First, we compared the expression levels of three UPR-related proteins, PERK, IRE1α, and ATF6, in three different hepatocytes, Huh7, HepG2, and HepG2-NTCP-C4 cells. As shown in Fig. 2A, rt269L HBV mainly induced PERK signaling, and the protein expression levels of p-PERK and p-eIF2α were significantly increased in rt269L-infected cells. Our immunofluorescence data also revealed that rt269L-infected cells increased p-eIF2α-positive signals (Fig. 2B). A similar result was obtained in the liver tissue of mice hydrodynamically infected with rt269L HBV, as indicated via immunohistochemistry (IHC) staining (Fig. 2C, Additional file 7: Fig. S3). Moreover, the ATF4 expression level was highly enhanced in rt269L-infected cells (Fig. 2D), indicating that rt269L induced PERK–eIF2α–ATF4 axis activation. As ATF4 is considered a key stress regulator [22], we further investigated the signal in genotype A HBV infection. To verify this, we used a plasmid and pHY92 vector containing a copy of the 1.1x-unit length HBV genome (genotype A HBV strain identical to GenBank AF305422) and performed a site-directed mutagenesis assay. Interestingly, enhanced ATF4 expression was observed in rt269L-infected cells in both genotypes A and C (Fig. 2E), which indicates that the HBV pol rt269L region is associated with PERK–eIF2α–ATF4 axis activation. Meanwhile, an XBP1 splicing assay was performed, and neither rt269L nor rt269I HBV induced IRE1α-sXBP signaling, but only thapsigargin (Tg) led to XBP splicing, showing spliced (269 bp) and unspliced (295 bp) forms (Fig. 2F, left panel). In addition, SYBR-based quantitative reverse transcription PCR (RT-qPCR) was performed, and the ATF4-encoding gene was profoundly upregulated in rt269L-infected cells, but no change was observed in the ATF6 and XBP1 genes (Fig. 2F, right panel). Altogether, rt269L type activated the PERK–eIF2α–ATF4 pathway, but rt269I type failed to induce the UPR under cellular stress in the in vitro or in vivo assays.

### rt269L genotype C HBV infection led to enhanced autophagy induction

**Fig. 3:**
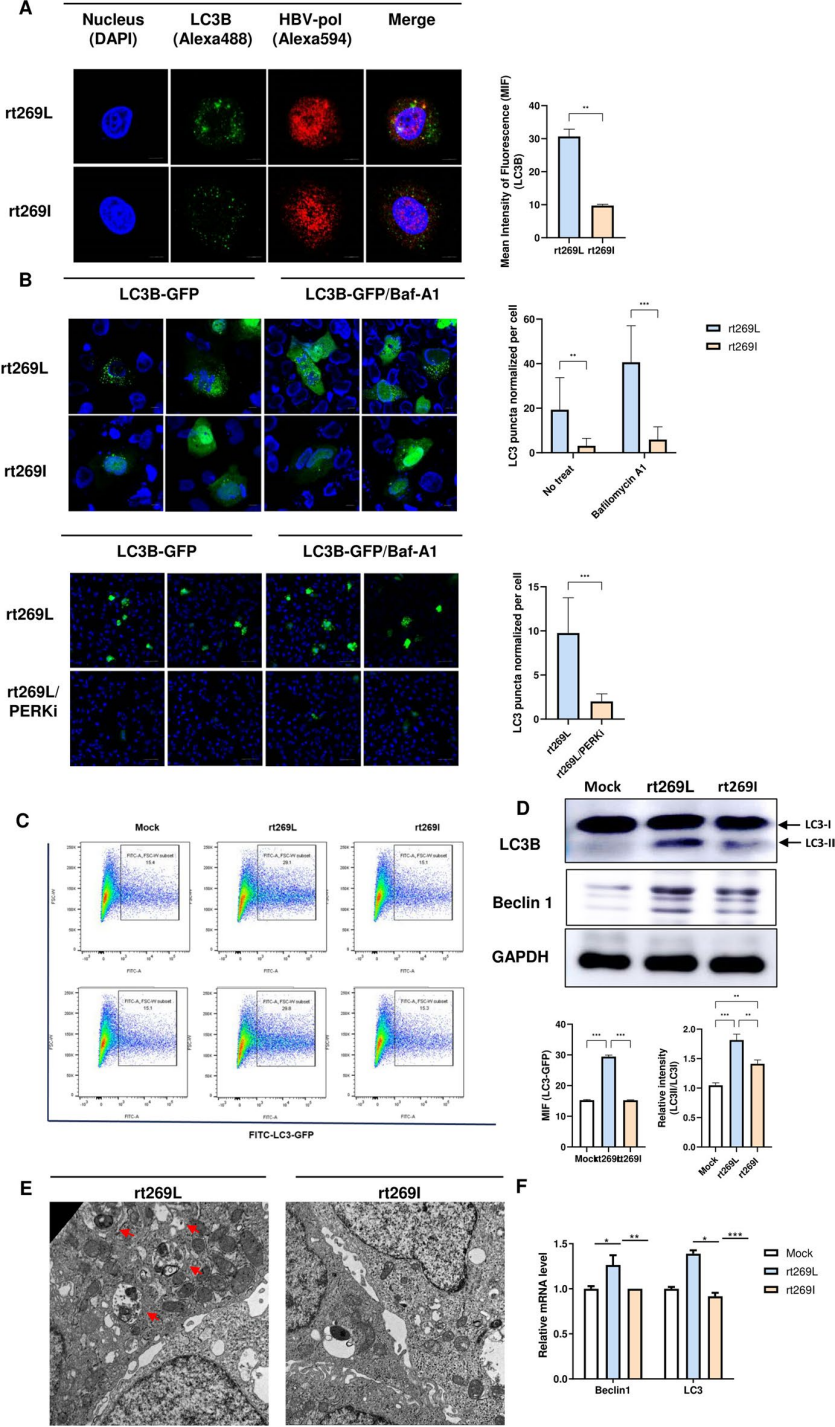
rt269L genotype C HBV infection led to enhanced autophagy induction. **A**, **B** Detection of autophagy induction by fluorescence microscopy. **A** HepG2 cells were transfected with the rt269L or rt269I vector. Cells were immunostained with antibodies against LC3B (green, Alexa 488) and HBV-pol (red, Alexa 594). The mean intensity of fluorescence (MIF) was analyzed. **B** Detection of LC3 puncta after transfection of the EGFP-LC3 vector. LC3 is recruited to autophagosomes, forming a punctate structure, as shown by green dots. The LC3-positive puncta in rt269L- or rt269I-transfected cells were analyzed in the absence or presence of bafilomycin A1 or a PERK inhibitor (GSK2656157, 10 µM). **C** EGFP-LC3 assay by flow cytometry. EGFP-LC3B-positive HepG2 cells cotransfected with mock, rt269L, or rt269I vectors were detected by flow cytometry. **D** Western blot analysis of the autophagy marker proteins LC3 and Beclin 1. The relative intensity of LC3II/LC3I staining was analyzed. **p < 0.01, ***p < 0.001. **E** Detection of autophagosomes and autolysosomes in HepG2-NTCP-C4 cells infected with rt269L or rt269I HBV virions, assessed by TEM. The arrow denotes autophagic vacuoles. Scale bar, 1 µm. **F** RT–qPCR analysis of Beclin1 and LC3 mRNA 10 h after HepG2-NTCP-C4 cell infection with PBS (Con), or rt269L or rt269I HBV virions. *p < 0.05, **p < 0.01, ***p < 0.001

ER stress can activate autophagy gene transcription through the PERK–eIF2α–ATF4 axis, and ATF4-mediated autophagy is known to be a potent cell survival mechanism [23, 24]. Because the rt269L type triggered PERK–eIF2α–ATF4 signaling, we investigated autophagy induction signals. First, we immunostained endogenous LC3 protein with an anti-LC3 antibody. As shown in Fig. 3A, rt269L infection exhibited a higher immunoreaction with anti-LC3 Ab than rt269I infection. Then, we quantified EGFP-LC3-labeled autophagosomes in the absence or presence of the lysosome inhibitor bafilomycin A1. As shown in Fig. 3B, rt269L led to increased LC3 puncta in a basal state, and bafilomycin A1 significantly enhanced the accumulation of EGFP-LC3 puncta. In contrast, rt269I infection showed low LC3 puncta formation in the basal state, and the number of puncta was not increased with bafilomycin A1. Next, we investigated whether autophagy induction depends on PERK signaling using a PERK inhibitor (GSK2656157). As we expected, PERK inhibitor treatment dramatically reduced the LC3 puncta count compared with that in rt269L-infected cells, suggesting that PERK signaling is a key mediator of autophagy induction in rt269L-infected cells. Our flow cytometry data consistently showed high autophagy induction in rt269L-infected cells (Fig. 3C, Additional file 8: Fig. S4), and immunoblot data indicated higher LC3-II and Beclin 1 protein expression in rt269L-infected cells than in rt269I-infected cells (Fig. 3D, Additional file 9: Fig. S5A). Furthermore, HepG2-NTCP-C4 cells infected with rt269L HBV virions formed many more autophagosomes and autolysosomes than rt269I-infected cells, as indicated by TEM (Fig. 3E). The mRNA expression of the autophagy markers Beclin 1 and LC3 was also detected by RT–qPCR, and the rt269L-infected cells showed higher autophagy gene transcription than the rt269I-infected cells (Fig. 3F).

**Fig. 4:**
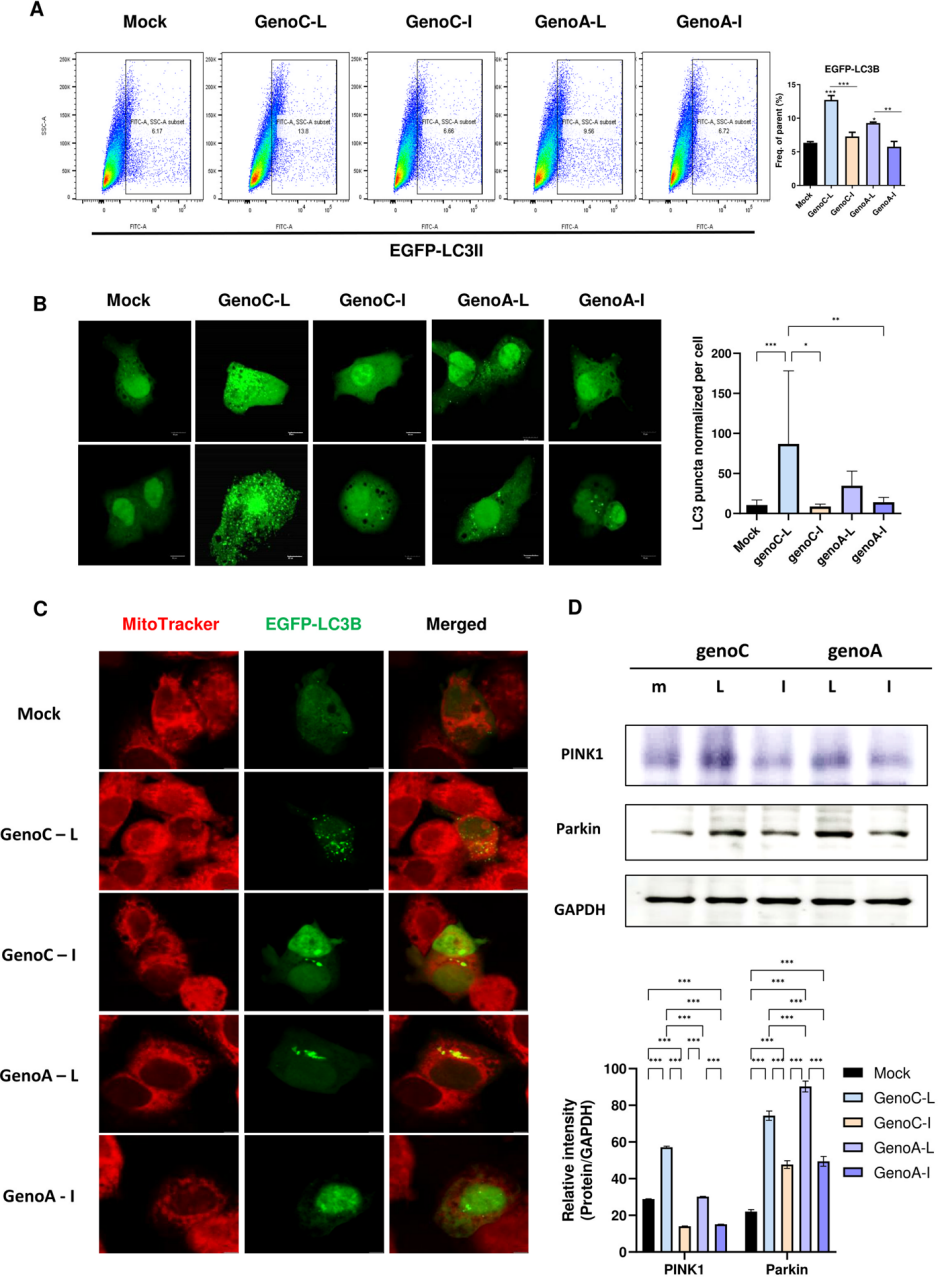
Comparison of autophagy/mitophagy induction in genotype A and C HBV infection. **A** EGFP-LC3 assay by flow cytometry. EGFP-LC3B-positive HepG2 cells cotransfected with mock, rt269L (genotype A or C), or rt269I (genotype A or C) vectors were detected by flow cytometry. **B** Detection of LC3 puncta after transfection of the EGFP-LC3 vector with genotype A or C vectors. LC3 is recruited to autophagosomes, forming a punctate structure, as shown by green dots. **C** Immunofluorescence for the colocalization of autophagosomes with mitochondria. The EGFP-LC3-expressing vector was cotransfected with HBV genome plasmids. Mitochondria were stained with MitoTracker deep red, and yellow dots represent colocalized EGFP-LC3-positive autophagosomes with red-labeled mitochondria. **D** Western blot analysis of the mitophagy marker protein PINK1. The relative intensity of PINK1/GAPDH staining was analyzed. **p < 0.01, ***p < 0.001

Next, we compared the autophagy induction patterns in genotypes A and C (Fig. 4A, B). Interestingly, genotype A rt269L, generated by site-directed mutagenesis, showed enhanced autophagy induction, suggesting that polymerase with rt269L distinct in genotype C infection could play a key role in PERK–eIF2α–ATF4 axis-mediated autophagy induction. Furthermore, we observed that HBx in rt269L strongly bound to PI3KC3, which induces autophagosome formation via PI3KC3/Beclin-2 activation [25, 26], in our immunoprecipitation assay (Additional file 9: Fig. S5B). Together, these data suggest that rt269L HBV infection significantly induces autophagy, mainly by activating PERK signaling, while rt269I HBV infection fails to activate autophagy.

### rt269L infection-induced mitophagy

For mitochondrial quality control, elimination of damaged organelles, such as mitochondria (mitophagy), is important to maintain normal cellular physiology [27]. To determine whether rt269L is involved in mitophagy, HepG2 cells were cotransfected with EGFP-LC3 and HBV plasmids and stained with MitoTracker red to analyze their colocalization. As shown in Fig. 4C and Additional file 10: Fig. S6, we observed that mitochondria in rt269L-infected cells were associated with EGFP LC3 puncta, and their colocalization rates were higher than those of the rt269I type. In addition, the mitophagy-associated protein markers PINK1 and Parkin were significantly increased in rt269L-infected cells (Fig. 4D), suggesting that rt269L-type HBV infection leads to mitophagy induction.

### Impaired autophagy in rt269I HBV infection induces caspase activation and cell death by triggering cytochrome c release

**Fig. 5:**
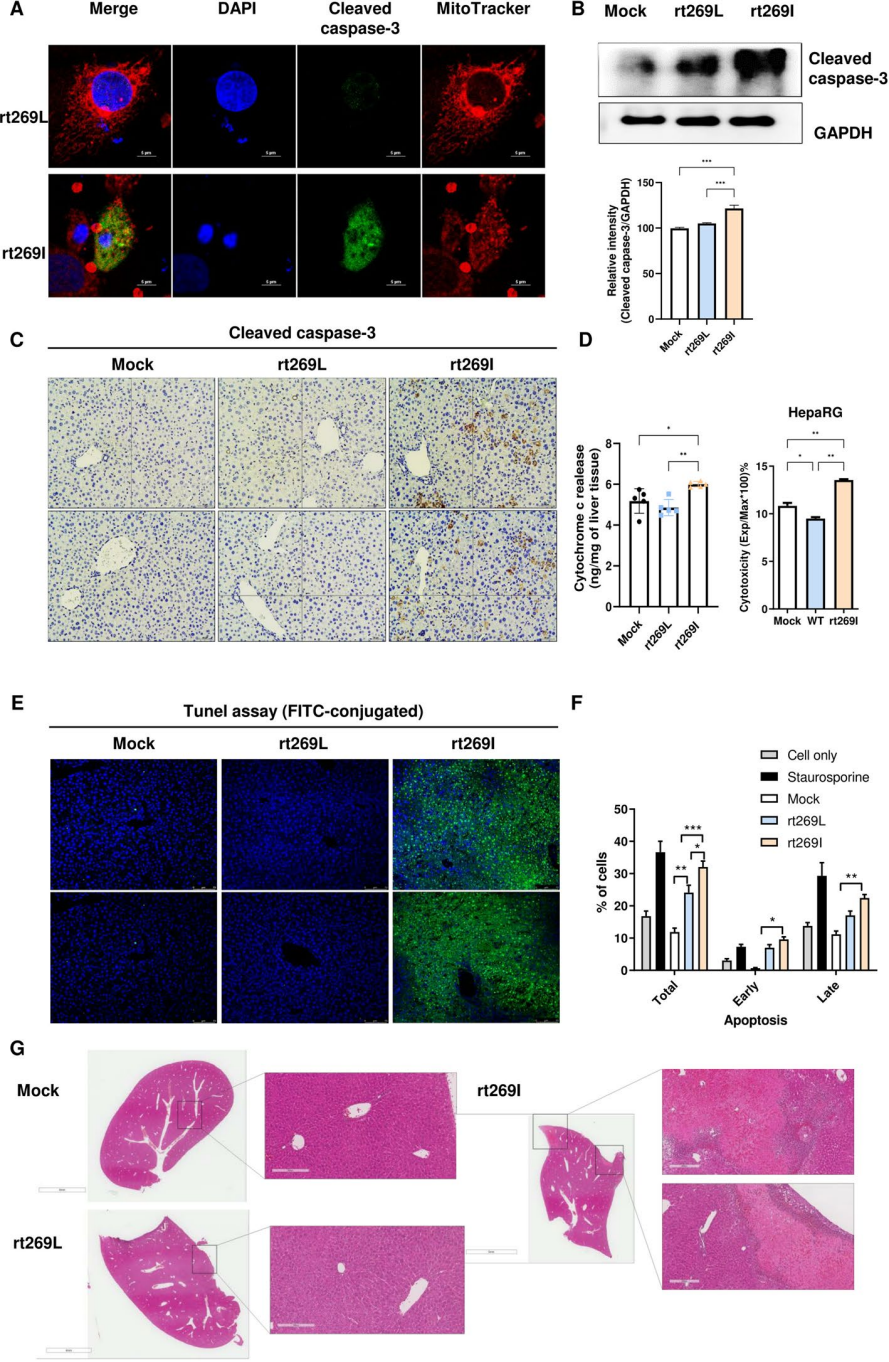
rt269I induces caspase activation and cell death by releasing cytochrome c. **A **Confocal images of HepG2 cells transfected with the rt269L or rt269I vector. Green fluorescence represents active (cleaved) caspase-3. Mitochondrial features were stained with MitoTracker, and nuclei were labeled with DAPI. Scale bar, 5 µm. **B** Western blot analysis of cleaved caspase-3 in HepG2 cells transfected with the rt269L or rt269I vector. The relative intensity of cleaved caspase-3 is compared with GAPDH. ***p < 0.001. **C** Immunohistochemistry analysis of cleaved caspase-3 expression in paraffin-embedded liver tissues (magnification 100 ×, *n* = 5 per group). **D** Left panel shows detection of cytochrome c in liver tissues of mice hydrodynamically injected with mock, rt269L, or rt269I vector and determined by ELISA. Right panel shows cytotoxicity levels measured in mock-, rt269L-, or rt269I-type HBV-infected HepaRG cells. **E** Cell death in liver tissues of mice hydrodynamically injected with mock, rt269L, or rt269I vector was detected by TUNEL assay (FITC-conjugated). **F** Detection of apoptotic HepG2 cells using Annexin V-FITC and 7-aminoactinomycin D (7-AAD) double-positive assays. Staurosporine (1 µM) was used to detect the positive control (Con), and the percentage of early or late apoptotic cells is indicated. **G** Mouse liver sections stained with H&E to analyze the liver injury and necrosis area among mouse groups hydrodynamically injected with mock, rt269L, or rt269I HBV. Magnification, 100×

Recent evidence further indicates that impaired autophagy results in increased apoptosis signals, rapid activation of caspase-3, and ultimately cell death [28, 29]. As rt269I-infected cells showed impaired autophagy induction, we hypothesized that rt269I disrupts cellular homeostasis and leads to cell death via caspase activation. To test this hypothesis, we measured active caspase-3 expression through in vitro and in vivo assays. First, our immunofluorescence data revealed that rt269I-infected cells exhibited the presence of caspase-3-positive compartments (Fig. 5A). In addition, the expression of cleaved caspase-3 protein was markedly increased in rt269I-infected cells (Fig. 5B). Then, we examined liver tissue samples from C57BL/6 mice hydrodynamically injected with rt269L or rt269I HBV to investigate cleaved caspase-3 expression via IHC. Consistently, rt269I-infected mice demonstrated a significant increase in active caspase-3-positive liver cells (Fig. 5C, Additional file 11: Fig. S7). Increased cytochrome c release was also detected in mouse liver tissue in the rt269I-infected group, and elevated cytotoxicity, as well as ROS, were shown in rt269I-infected HepaRG cells and HepG2 cells, respectively (Fig. 5D, Additional file 12: Fig. S8).

Furthermore, a TUNEL assay exhibited clear apoptotic features in the liver tissue of rt269I-infected mice (Figs. 5E, Additional file 13: Fig. S9), and flow cytometry analyses showed increased apoptosis of hepatocytes transfected with rt269I HBV (Fig. 5F). Moreover, hematoxylin and eosin (H&E) staining found that rt269I-infected mouse liver tissues exhibited many necrotic hepatocytes and denucleated cells (Fig. 5G). Altogether, impaired autophagy in rt269I infection made cells vulnerable to further elevation of ROS and induced cell death through caspase-3 activation.

### rt269L infection affects HBx protein levels

**Fig. 6:**
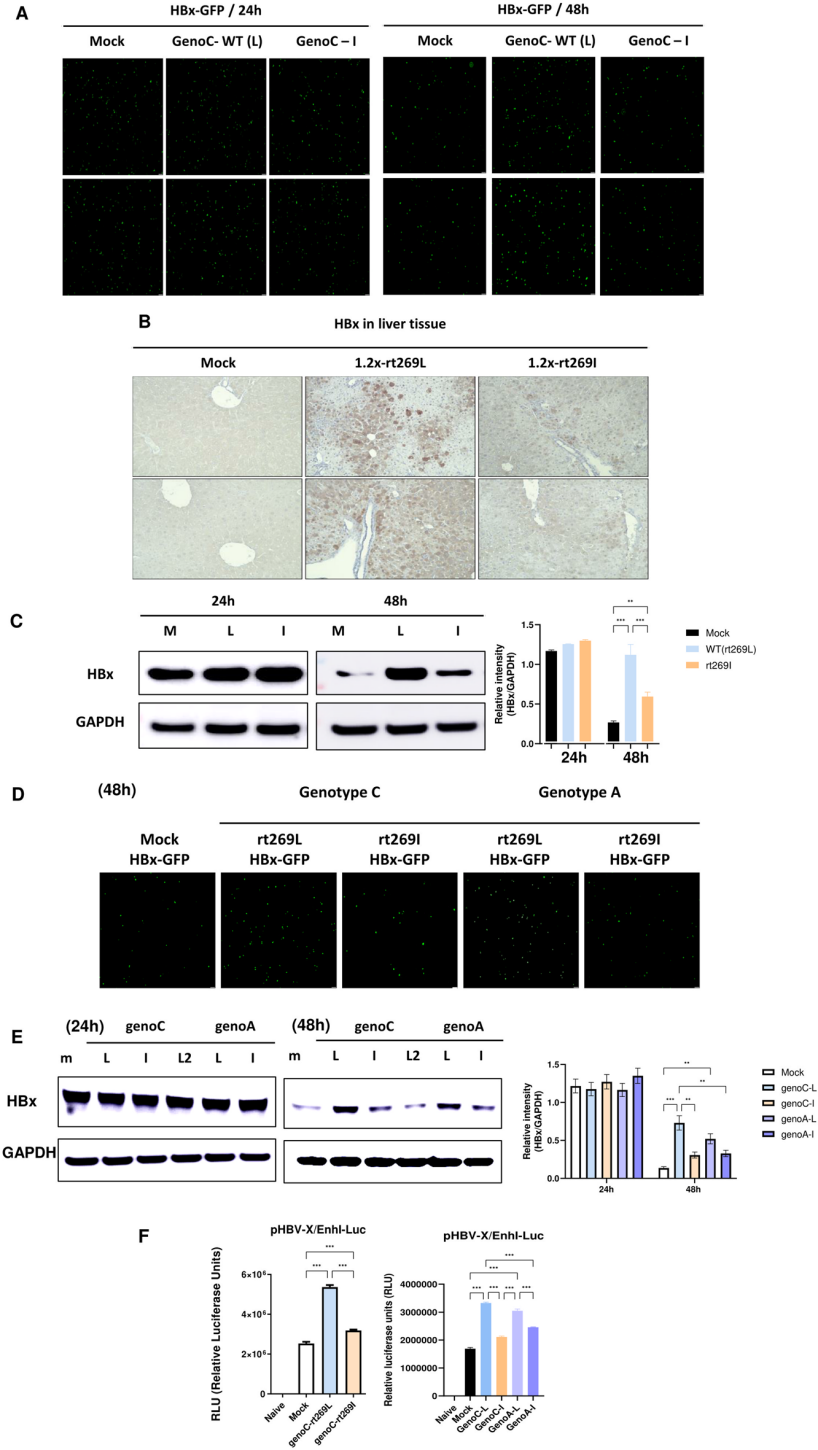
rt269L leads to increased HBx protein stability. **A** Immunofluorescence images of HBx-GFP expression in HepG2 cells cotransfected with mock, rt269L, and rt269I whole genome plasmids. **B** Immunohistochemical (IHC) analysis of HBx expression in paraffin-embedded liver tissues (magnification 100 ×, *n* = 5 per group). **C** Western blot analysis of HBx-GFP in HepG2 cells cotransfected with mock, rt269L, or rt269I vector. **D** Immunofluorescence images of HBx-GFP expression in HepG2 cells cotransfected with mock, rt269L (genotype A or C), and rt269I (genotype A or C) whole genome plasmids. **E** Western blot analysis of HBx-GFP in HepG2 cells cotransfected with mock, rt269L (genotype A or C), or rt269I (genotype A or C) vector. **F** Luciferase reporter assay with the pHBV-X/EnhI-Luc construct composed of the X promoter region and enhancer I at 48 h. The relative intensity of HBx/GAPDH staining was analyzed. **p < 0.01, ***p < 0.001

We sought to further investigate the underlying mechanisms of the improved mitochondrial functionality and high autophagy induction in rt269L HBV infection. As HBV X protein (HBx) has known to play a central role in resisting cell death via autophagy induction [30–32], we hypothesized that the high capacity to maintain HBx protein in rt269L would be an upstream signaling pathway explaining several characteristics found in HBV genotype C2 infection. First, we tested whether rt269L could affect the HBx protein level. HepG2 cells were cotransfected with HBx-GFP and rt269L or rt269I whole genome plasmids. As shown in Fig. 6A, rt269L significantly increased HBx-GFP expression levels at 48 h postinfection. A similar result was obtained in the liver tissue of mice hydrodynamically infected with rt269L HBV (Fig. 6B). In addition, rt269L significantly increased HBx protein levels at 48 h posttransfection in HepG2 cells (Fig. 6C). Interestingly, a similar result was observed in genotype A rt269L, generated by site-directed mutagenesis (Fig. 6D, E). Moreover, HBx protein is known to exert an anti-apoptotic effect via activation of Akt signaling [33, 34], and our data also showed that rt269L infection activated phospho-Akt and phospho-PI3K (Additional file 14: Fig. S10, Additional file 15: S11).

### Enhanced HBx expression in rt269L activates Enhancer I promoter

Enhancer I (EnhI) is located upstream of and overlaps the X promoter, and it influences the transcription of the HBx gene [35]. To investigate the change in EnhI promoter expression in rt269L, we performed a luciferase reporter assay that measured HBx/EnhI promoter activity [36]. As shown in Fig. 6F, rt269L-cotransfected cells revealed enhanced activation of the HBx/EnhI promoter. Similarly, increased transcription levels of the HBx/EnhI promoter were also shown in rt269L-transfected cells in the genotype A group (Fig. 6F, right panel).

### rt269L leads to increased HBx protein stability

**Fig. 7:**
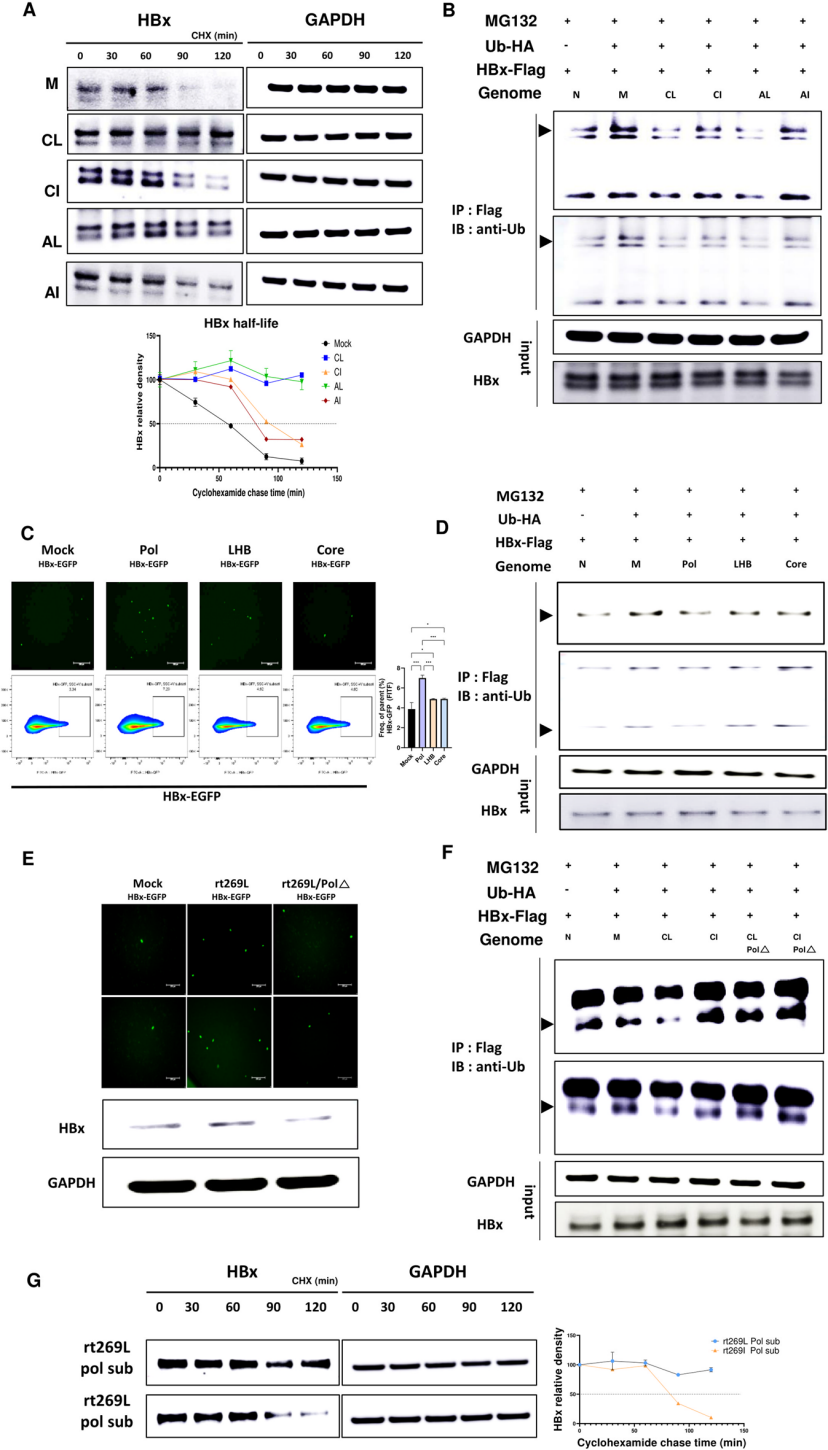
rt269L attenuates HBx degradation through ubiquitination of HBx. **A** HepG2 cells were cotransfected with pHBx-GFP and mock, rt269L (genotype A or C), or rt269I (genotype A or C)-containing plasmids. Forty-eight hours posttransfection, the cells were treated with 100 µg/ml cyclohexamide to inhibit de novo translation. Cells were lysed at the indicated timepoints and analyzed by western blotting with anti-HBx or anti-GAPDH antibody. **B** HepG2 cells were cotransfected with pHBx-FLAG in combination with mock, rt269L (genotypes A and C), and rt269I (genotypes A and C) containing plasmids. Cells were treated with 20 µM MG132 at 42 h posttransfection for 6 h. Total cell lysates were immunoprecipitated using an anti-FLAG antibody and analyzed by western blotting using an anti-Ubiquitin antibody. **C** Level of HBx-GFP expression of a HBV subgenome-containing plasmid cotransfected into HepG2 cells, as indicated by fluorescence and flow cytometry assays. **D** HepG2 cells were cotransfected with pHBx-FLAG in combination with mock, Pol-, LHB-, and Core ORF-containing plasmids. Total cell lysates were immunoprecipitated using an anti-FLAG antibody and analyzed by western blotting using an anti-Ubiquitin antibody. **E** Detection of HBx-GFP protein expression in mock, rt269L, or rt269L/Pol∆ cotransfected HepG2 cells, as shown in fluorescence and western blot assays. **F** HepG2 cells were cotransfected with pHBx-FLAG in combination with mock and Pol-deleted rt269L/I plasmids. Total cell lysates were immunoprecipitated using an anti-FLAG antibody and analyzed by western blotting using an anti-Ubiquitin antibody. **G** A cycloheximide chase experiment with rt269L- or rt269I-containing Pol subgenome-carrying plasmid-transfected cells

To verify the increase in HBx protein levels in rt269L infection, we performed a cycloheximide chase experiment to determine whether rt269L altered HBx stability. As shown in Fig. 7A, rt269L transfection significantly extended the half-life of HBx protein from approximately 60 min to 120 min in both the genotype A and C groups. These results suggest that rt269L HBV infection leads to enhanced HBx protein stability.

### rt269L attenuates HBx degradation through ubiquitination of HBx

HBx turnover can be processed by both ubiquitin-dependent and ubiquitin-independent proteasomal steps [37]. To verify the underlying mechanism of the enhanced HBx stability in rt269L-infection, we performed a ubiquitination assay by detecting the ubiquitination of HBx proteins with immunoprecipitation. As shown in Fig. 7B, rt269L infection, in both genotypes A and C, revealed diminished ubiquitination of HBx compared with rt269I infection. Then, to clarify the components in the HBV protein that are critical to the stability of the HBx protein, we cloned the Pol (a polymerase), LHB (a large surface protein containing preS1, preS2, and small S), and Core ORFs into expression plasmids, and coexpressed each subgenome-containing vector with pGFP-HBx. As shown in Fig. 7C, the Pol subgenome group showed significantly increased GFP-HBx expression levels both in fluorescence microscopy images and flow cytometry assays compared with those in the other subgenome groups. In addition, the Pol subgenome-containing vector altered the ubiquitination rate of HBx, in contrast to the other subgenome-containing vectors (Fig. 7D), indicating that the HBV Pol genome and the proteins it encodes are critical for HBx production and ubiquitination. Then, we confirmed that HBx induction is stimulated in a Pol-dependent manner. We generated an rt269L/Pol∆ plasmid (Additional file 16: Fig. S12), which does not express Pol, via site-directed mutagenesis and observed HBx expression when the plasmid was coexpressed with pGFP-HBx. As shown in Fig. 7E, the rt269L/Pol∆ group showed a reduced GFP-HBx expression level, which was increased in the rt269L group, as indicated in both the fluorescence microscopy and western blot images.

Furthermore, decreased ubiquitination of the HBx protein in the rt269L group was also found (Fig. 7F) when the Pol-rt269 region was deleted by mutagenesis (Additional file 17: Fig. S13), and HBx protein stability was measured in a cycloheximide chase experiment after a rt269L-containing Pol subgenome-carrying vector was transfected into HepG2 cells (Fig. 7G). Together, these data indicated a significant role for HBV Pol with rt269L in maintaining HBx protein stability via deubiquitination.

### Autophagy induction and UPR responses were activated in an HBx-dependent manner

**Fig. 8:**
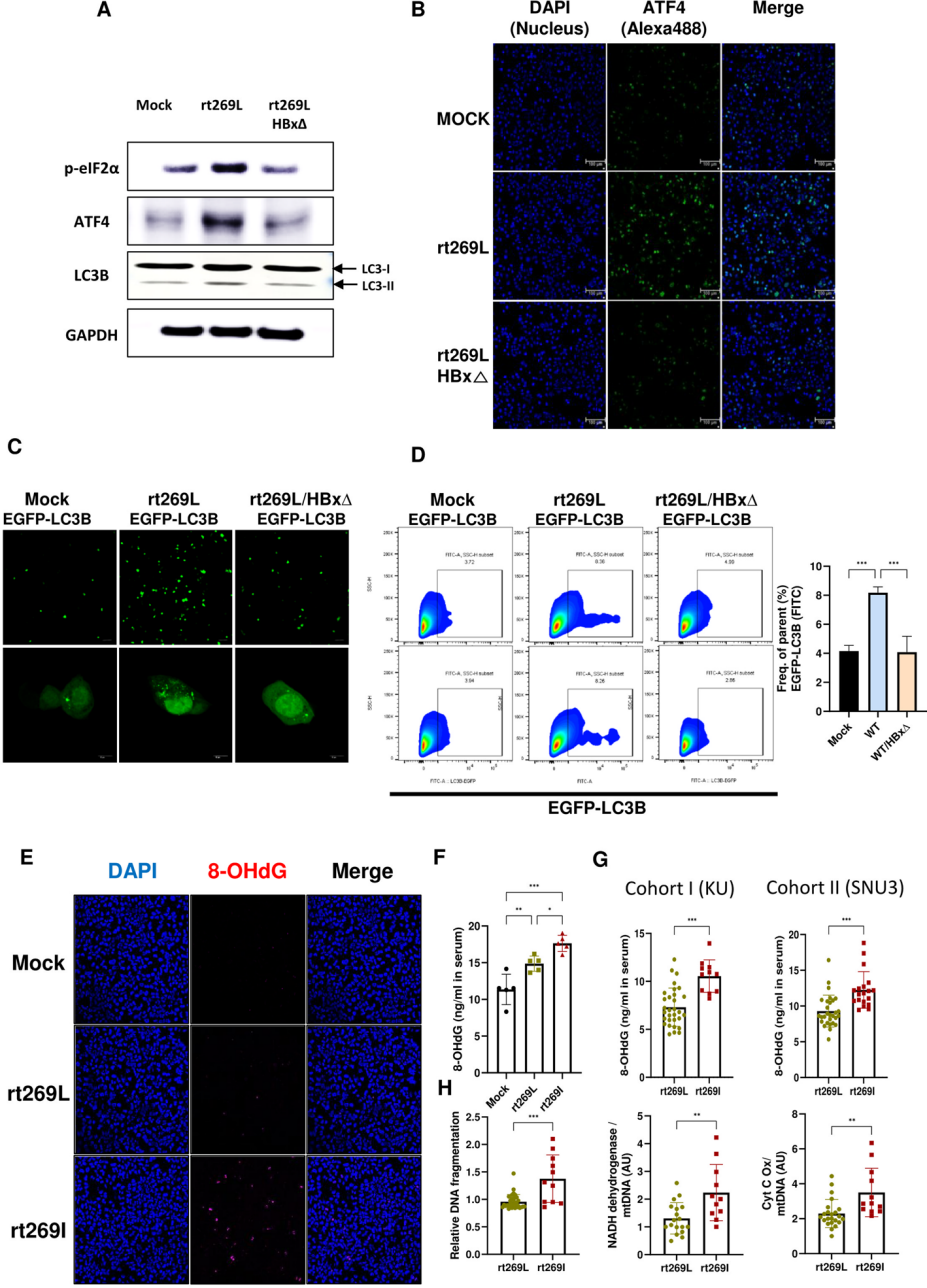
Elevated hepatic oxidative damage in rt269I HBV infection and its clinical implications. **A** Western blot analysis of p-eIF2α, ATF4, LC3B, and GAPDH in HepG2 cells transfected with mock, rt269L, or rt269L/HBx∆ vector. **B** ATF4 expression determined by immunofluorescence assay. **C, D** Measurement of LC3 puncta after cotransfection of the EGFP-LC3 vector with mock, rt269L-, or rt269L/HBx∆-containing plasmids, as determined by immunofluorescence and flow cytometry assays. LC3 is recruited to autophagosomes, forming a punctate structure, as shown by green dots. **E** Formation of 8-OHdG in hepatocytes transfected with rt269I. HBV representative immunofluorescence images showing 8-OHdG in HepG2 cells transiently transfected with the mock, rt269L, or rt269I vector. Nuclei were stained with DAPI (blue). 8-Hydroxy-2′-deoxyguanosine (8-OHdG). **F** Detection of 8-OHdG release in serum in mice hydrodynamically infected with mock, rt269L, or rt269I vectors. **G** Measurement of the 8-OHdG release level in patient serum collected from two independent cohorts (KU and SNU3). **H** Mitochondrial damage biomarkers in patient serum. Detection of nuclear DNA (nDNA) fragmentation release in serum detected by ELISA and mitochondrial DNA release in serum detected by RT–qPCR. Scatter dot plots were generated using GraphPad Prism 9.0 software (GraphPad, La Jolla, CA, USA). *p < 0.05, **p < 0.01, ***p < 0.001

As we verified increased HBx stability in rt269L HBV infection, we examined whether autophagy induction and PERK–eIF2α–ATF4 signaling were stimulated in an HBx-dependent manner. We generated the rt269L/HBx∆ plasmid by mutagenesis, which does not express HBx protein, and compared the protein expression levels of p-eIF2α, ATF4, and LC3B in rt269L- and rt269L/HBx∆ transfected HepG2 cells. As shown in Fig. 8A, the increased protein expression levels of p-eIF2α, ATF4, and LC3B in rt269L cells were diminished in rt269L/HBx∆ transfected cells. A similar result was shown in immunofluorescence images, in which ATF4 expression in rt269L-transfected hepG2 cells was increased, whereas that in rt269L/HBx∆-transfected cells was reduced (Fig. 8B). In addition, rt269L/HBx∆ dramatically reduced the number of EGFP-LC3B-positive cells, as well as the punctuation, indicating that enhanced HBx protein stability in rt269L is an upstream mediator of autophagy induction and UPR responses (Fig. 8C, D).

### Elevated hepatic oxidative damage in rt269I HBV infection and its clinical implications

In contrast to rt269L, rt269I infection failed to control mitochondrial quality and showed impaired autophagy induction, which could lead to hepatic oxidative damage. 8-OHdG is a critical biomarker of oxidative stress and chronic liver disease progression [38]. We observed elevated 8-OHdG levels in the immunofluorescence assay in HepG2 cells transiently transfected with the rt269I vector (Fig. 8E). Similarly, rt269I-infected mice secreted approximately 1.5-fold higher levels of 8-OHdG compared with the rt269L-injected group (Fig. 8F). We then examined 8-OHdG levels in patients’ sera from two different Korean cohorts, and rt269I-infected patients showed higher 8-OHdG release in their serum than rt269L-infected patients (Fig. 8G). Then, we also tested mitochondrial biomarkers in patient sera from the cohorts. The detection of nDNA fragmentation, and mitochondrial DNA and proteins in serum clinically represents mitochondrial damage, liver inflammation, and liver injury [39]. As shown in Fig. 8H, patients infected with rt269I HBV showed more nDNA fragments released in their serum than patients with rt269L HBV infection. Additionally, serum mtDNA release (indicating NADH dehydrogenase activity and cytochrome c oxidation) was 1.5- to 2-fold higher in patients with rt269I infection than in patients with rt269L infection (Fig. 8H). These data suggest that the mitochondrial damage in patients with rt269I infection may be more severe than that in patients with rt269L infection.

## Discussion

Genotype C, particularly subgenotype C2, is responsible for most chronic infections in HBV-endemic East Asian countries, including China, Japan, and South Korea. This genotype is most closely associated with an increased risk of liver cancer (LC) and HCC, with characteristics including high HBV DNA levels in serum and an elevated tendency for chronicity and mutation [40]. However, the mechanisms underlying these characteristic traits of genotype C infection remain largely unknown. Of note, there are two distinct polymorphisms based on the HBV Pol-269 site, rt269L and rt269I types, in chronic patients with HBV genotype C infection [17]. Recently, we reported that the rt269I HBV genotype C type versus rt269L was more significantly associated with HBeAg-negative seroconversion, liver disease progression, and high mutation rates [17, 18]. In this study, we hypothesized that rt269L infection in the HBV RT region, evident only in genotype C, may play prominent roles in some distinct traits in patients infected with genotype C, including higher infectivity or longer duration of HBeAg positive stage. We sought to explore this issue, focusing on differences in mitochondrial functionality and associated upstream signaling, including ER stress and autophagy induction, between rt269L and rt269I types.

First, we demonstrated that the rt269L mutation leads to increased HBx protein stability, via deubiquitination, compared with the rt269I mutation (Fig. 7). Furthermore, this trend is also true in genotype A infection: the genotype A variant with the rt269L mutation in polymerase via mutagenesis also led to enhanced HBx protein stability compared with the wild-type (Fig. 7). This finding suggests a significant role of HBV Pol with rt269L in HBx protein stability, irrespective of genotype. The HBx protein has multifaceted roles in HBV-related pathogenesis, including HCC or LC progression, as well as viral replication [33, 41, 42]. Of note, it has also been reported to play a very pivotal role in mitochondrial quality control via induction of autophagy/mitophagy [31, 43] Therefore, the enhanced autophagy/mitophagy induction-mediated improved mitochondrial dynamics and bioenergetics [44] found in rt269L infections in this study (Figs. 1, 3) may be due to enhanced stability of HBx protein, resulting in increased survival of infected hepatocytes, which could contribute to increased HBV replication, persistent infection, or longer duration of HBeAg positive phase, all distinct traits of genotype C infections. Moreover, HBx protein has been reported to exert an anti-apoptotic effect in infected hepatocytes via activation of Akt signaling [30, 34]. Our data also showed that rt269L infection leads to increased Akt-PI3K activation and exerts an anti-apoptotic effect (Additional file 14: Fig. S10, Additional file 15: Fig. S11), suggesting a possible contribution of rt269L infection to the increased HCC risk of genotype C infections.

PERK signaling plays a significant role in the maintenance of mitochondrial homeostasis, mainly via the PERK–eIF2α–ATF4 axis at the interface of the ER and mitochondria during ER stress, alleviating the stress responses of both organelles [13]. Of these, ATF4, a key player in the integrating stress response (ISR), can transcriptionally regulate more than a dozen ATG genes via activation of C/EBP homologous protein (CHOP), a transcription factor, providing a substantial link between autophagy and the UPR [45]. Indeed, our data indicated that rt269L infection can trigger the PERK–eIF2α–ATF4 axis (Fig. 2), in turn contributing to improved mitochondrial dynamics and energetics in infected hepatocytes via autophagy/mitophagy induction (Fig. 1, 3, 4). In contrast, the rt269I variant failed to trigger PERK signaling during infection, resulting in severe mitochondrial dysfunction via failure of autophagy induction (Figs. 1, 3, 4).

Notably, dual outcomes of PERK signaling have been described, with PERK playing roles in both cell survival and apoptosis [46, 47]. Genotype C rt269L infection seemed to leverage PERK signaling to promote viral survival and proliferation. We believe that rt269L infection leads to cellular survival and stable replication by increasing mitochondrial functionality via induction of PERK signaling-mediated autophagy, contributing to the high infectivity found in patients with genotype C infections. In contrast, the rt269I HBV variant, with impaired PERK signaling, leads to altered mitochondrial functionality, contributing to severe hepatic disorders and liver disease progression in patients with genotype C infections. Furthermore, the clinical severity caused by mitochondrial dysfunction and hepatic disorders observed after rt269I infection was also assessed by evaluating the expression of several biomarkers in CHB patient serum (Fig. 8G, H). As mitochondrial contents and DNA fragments detected in plasma are considered clear markers of mitochondrial injury and DNA fragmentation, respectively, particularly in necrotic cells [48], higher levels of 8-OHdG, DNA fragments, and mtDNA release in rt269I infections (Fig. 8G, H) found in Korean patient sera further support our hypothesis associated with poor clinical outcomes for patients with genotype C2 rt269I infection.

Taking these findings together, we verified that HBx stability and cellular quality control mediated via autophagy in the rt269L (WT) type group may indicate a major reason for the clinical features of genotype C infection, such as high replication and infection rates, sustained cccDNA, and a lower response to IFN therapy. Meanwhile, as the high infectivity of rt269L was retained, leading to chronic infection, the rt269I-type variant developed and showed increasingly higher mutation rates, leading to liver disease progression. Functional differences at the molecular level between rt269L and rt269I in HBV genotype C infection need to be further characterized in the future, which could provide new insight into developing a novel therapeutic strategy for HBV genotype C infection.

## Conclusions

Our data showed that the rt269L type presented exclusively in HBV genotype C infection, versus rt269I being common in other genotypes, and can lead to improved mitochondrial dynamics or bioenergetics, which is mainly due to autophagy induction via activation of the PERK–eIF2α–ATF4 axis in an HBx protein-dependent manner. This suggests that rt269L type predominating in genotype C endemic areas could at least partly contribute to some distinct traits of genotype C infection, such as higher infectivity or longer duration of HBeAg positive stage. In contrast, the rt269I variant in HBV genotype C infection is significantly associated with mitochondrial dysfunction and impaired autophagy due to impaired PERK signaling, resulting in enhanced ROS production and apoptosis in infected hepatocytes. CHB patients infected with the rt269I variant of genotype C likely have increased risks of LC caused by hepatic disorder, ROS leakage, ATP deficiency, and severe DNA damage compared with those with rt269L HBV (Graphical abstract).

## Supplementary Information

**Additional file 1: Table S1.** Comparison of clinical factors between two variants in the rt269 codon.

**Additional file 2: Table S2.** Comparison of clinical factors between two variants in the rt269 codon.

**Additional file 3: Table S3.** PCR primers used in this study.

**Additional file 4: Table S4.** List of Antibodies.

**Additional file 5: Figure S1.** Related to Fig. 1., rt269L type showed improved mitochondrial maintenance, with high rate of functional mitochondria and biogenesis. Confocal microscopy images showing HepG2 cells transfected with mock, rt269L, or rt269I HBV followed by TMRM staining. Mitochondrial outer membrane permeabilization (MOMP) and ΔΨm were observed. Nuclei were stained with DAPI (blue). Scale bar, 50 μm

**Additional file 6: Figure S2. A** WT (rt269L) HBV induced higher transcription of three different mtDNA genes than in rt269I HBV infection. Mitochondrial copy numbers were calculated based on RT–qPCR results obtained with three different mtDNA primer sets. The results were evaluated for statistical significance by one-way ANOVA with Tukey’s post hoc test or *t*-test. Differences were considered significant when *p < 0.05, **p < 0.01, or ***p < 0.001. **B**. Protein expression levels were analyzed by western blotting with antibodies against PGC1α and GAPDH. The relative intensity compared with GAPDH was analyzed. *p < 0.05, **p < 0.01, ***p < 0.001

**Additional file 7: Figure S3.** WT (rt269L) HBV induces ER stress and activates the PERK-mediated pathway in the unfolded protein response (UPR). Immunohistochemical analysis of p-eIF2α expression in paraffin-embedded liver tissues (magnification 100×, *n* = 5 per group)

**Additional file 8: Figure S4.** EGFP-LC3 assay with flow cytometry. EGFP-LC3B-positive HepG2 cells cotransfected with mock, rt269L, or rt269I HBV were detected by flow cytometry

**Additional file 9: Figure S5.** **A** Impaired autophagy in rt269I HBV infection western blots showing the autophagy marker protein LC3. **B** HBx in rt269L strongly interact with PI3KC3 (VPS34). HBx-flag plasmid was cotransfected with mock, genoC-L, genoC-I, genoA-L, or genoC-I plasmid in HepG2 cells, as indicated. At 42 h posttransfection, cells were extracted and the cell lysates were subjected to IP with anti-Flag Ab followed by IB using anti-PI3KC3 Ab

**Additional file 10: Figure S6.** Immunofluorescence for the colocalization of autophagosomes with mitochondria. EGFP-LC3-expressing vector was cotransfected with HBV genome plasmids. Mitochondria were stained with MitoTracker deep red, and yellow dots represent colocalized EGFP-LC3-positive autophagosomes with red labeled mitochondria

**Additional file 11: Figure S7.** rt269I HBV induces caspase activation and cell death Immunohistochemistry analysis of cleaved caspase-3 expression in paraffin-embedded liver tissues (magnification 100×, *n* = 5 per group)

**Additional file 12. Figure S8. **Detection of intracellular reactive oxygen species in hepatocytes transfected with rt269L or rt269I HBV. Reactive oxygen species (ROS) were detected with a CM-H2DCFDA fluorescence probe in hepatocytes transfected with the rt269L or rt269I HBV vector. H_2_O_2_ was used as the positive control for ROS induction

**Additional file 13: Figure S9.** Cell death in liver tissues of mice hydrodynamically injected with the mock, rt269L, or rt269I vector was detected by TUNEL assay (FITC-conjugated). Nuclei were stained with DAPI (blue)

**Additional file 14: Figure S10. **rt269L-type HBV infection, in both genotype C and A, activated phospho-Akt and phospho-PI3K signals. Western blot analysis of the phospho-PI3K and phosphor-Akt, and GAPDH. The relative intensity was analyzed. **p < 0.01, ***p < 0.001

**Additional file 15: Figure S11.** rt269L-type HBV infection activated phospho-mTOR and phospho-PI3K signals in HBx protein dependent manner. Western blot analysis of the phospho-PI3K and phospho-mTOR, and GAPDH. The relative intensity was analyzed. **p < 0.01, ***p < 0.001

**Additional file 16: Figure S12.** Site-directed mutagenesis to introduce a stop codon downstream of the polymerase start region

**Additional file 17. Figure S13.** Site-directed mutagenesis to introduce a stop codon upstream of the rt269 region. A stop codon was inserted at 49 bp (887 bp) upstream of rt269 region (936 bp) by site-directed point mutation. Conversion from TAT to TAA (stop) prevented the translation of full-length Pol-RT269 region

**Additional file 18.** Materials and methods

## Data Availability

All data generated or analyzed during this study are included in this article (and its additional files).

## Abbreviations

mtDNA: Mitochondrial DNA
HBeAg: Hepatitis B e antigen
HCC: Hepatocellular carcinoma
8-OHdG: 8-Hydroxy-2′-deoxyguanosine
PERK: Protein kinase R (PKR)-like ER kinase
ATF6: Activating transcription factor 6
IRE1α: Inositol-requiring enzyme 1 alpha
LC3: Microtubule-associated protein 1 light chain 3 beta
UPR: Unfolded protein response
