# Supplementary material for: rt269L-Type hepatitis B virus (HBV) in genotype C infection leads to improved mitochondrial dynamics via the PERK–eIF2α–ATF4 axis in an HBx protein-dependent manner
Source: Cell Mol Biol Lett. 2023 Mar 30;28:26. doi: 10.1186/s11658-023-00440-1 (PMC10064691; doi:10.1186/s11658-023-00440-1)
Supplement: Supplementary file 1 — Additional file 1: Table S1. Comparison of clinical factors between two variants in the rt269 codon. [file 11658_2023_440_MOESM1_ESM.pdf]

Supplementary Table S1.

KU (n = 90)	rt269L (n=59)	rt269I (n=31)	p-value
Sex (M/F), n (%)	34/25 (57.6/42.4)	19/12 (61.3/38.7)	0.7678
Age (years)	43 [18,73]	44.5 [22,73]	0.3638
HBeAg status (positive, %)	40 (67.8)	17 (54.8)	0.0393*
HBV DNA (log_{10} IU/mL)	6.73 [2.78,9.58]	5.99 [3.23,9.43]	0.0147*
qHBsAg (log_{10} IU/mL)	3.7 [1.5,5.1]	3.59 [1.4,4.98]	0.1298
AST (IU/L)	59 [16,686]	60 [19,850]	0.8185
ALT (IU/L)	62 [12,659]	63 [10,425]	0.5141
AST/ALT	0.88 [0.34,5.69]	1 [0.35,8.95]	0.1374
Total bilirubin (mg/dL)	0.8 [0.2,6]	1 [0.3,7]	0.0037**
Platelet count ($x10^3/mm^3$)	171 [23,328]	143 [36,350]	0.1839
Albumin (g/dL)	4.1 [2.3,5]	4.3 [2.3,4.8]	0.3845
Prothrombin time (INR)	1.08 [0.87,1.72]	1.11 [0.88,1.79]	0.5236
Presence of LC (no/yes)	39/20 (66.1/33.9)	18/13 (58.1/41.9)	0.2340
Presence of HCC (no/yes)	50/9 (84.7/15.3)	23/8 (74.2/25.8)	0.0734
FIB-4 score	1.54 [0.36,68.71]	2.36 [0.31,26.03]	0.1456

Table S1 Comparison of clinical factors between two variants in the rt269 codon ALT, alanine aminotransferase; AST, aspartate aminotransferase; HBeAg, hepatitis B e antigen; HBsAg, hepatitis B s antigen; HBV DNA, hepatitis B virus DNA; INR, international normalized ratio; qHBsAg, quantitative HBsAg levels; LC, liver cirrhosis; HCC, hepatocellular carcinoma; FIB-4, fibrosis-4. Data represent the frequency and percentage or Med [Min, Max], *p<0.05, **p<0.01, ***P<0.001.
