# Supplementary material for: rt269L-Type hepatitis B virus (HBV) in genotype C infection leads to improved mitochondrial dynamics via the PERK–eIF2α–ATF4 axis in an HBx protein-dependent manner
Source: Cell Mol Biol Lett. 2023 Mar 30;28:26. doi: 10.1186/s11658-023-00440-1 (PMC10064691; doi:10.1186/s11658-023-00440-1)
Supplement: Supplementary file 2 — Additional file 2: Table S2. Comparison of clinical factors between two variants in the rt269 codon. [file 11658_2023_440_MOESM2_ESM.pdf]

Supplementary Table S2.

SNU3 (n = 97)	**rt269L** (n=68)	**rt269I** (n=24)	p-value
Sex (M/F), n (%)	54/14	22/2	0.17
	(79.4/20.1)	(91.7/8.3)	
Age (years)	50.1 [22,72]	53.3 [22,95]	0.25
AST (IU/L)	71.2	64.9	0.83
ALT (IU/L)	65.7 [10,652]	59.4 [10,425]	0.80
AST/ALT	1.10 [0.5,2.41]	1.15 [0.35,8.95]	0.68
Total bilirubin (mg/dL)	1.68	2.67	0.27
Albumin (g/dL)	3.82	3.74	0.64
Globulin (g/dL)	3.36	3.38	0.91
Albumin/Globulin	1.14	1.13	0.90
γ-GTP	44.7	47.3	0.76
Presence of LC (no/yes)	47/21	14/10	0.34
	(69.1/30.9)	(58.3/41.7)	
Presence of HCC (no/yes)	53/15	18/6	0.76
	(77.9/22.1)	(75.0/25.0)	

Table S2 Comparison of clinical factors between two variants in the rt269 codon ALT, alanine aminotransferase; AST, aspartate aminotransferase; γ-GTP, gamma-glutamyl transpeptidase; LC, liver cirrhosis; HCC, hepatocellular carcinoma;. Data represent the frequency and percentage or Med [Min, Max], *p<0.05, **p<0.01, ***P<0.001.
