# Supplementary material for: rt269L-Type hepatitis B virus (HBV) in genotype C infection leads to improved mitochondrial dynamics via the PERK–eIF2α–ATF4 axis in an HBx protein-dependent manner
Source: Cell Mol Biol Lett. 2023 Mar 30;28:26. doi: 10.1186/s11658-023-00440-1 (PMC10064691; doi:10.1186/s11658-023-00440-1)
Supplement: Supplementary file 3 — Additional file 3: Table S3. PCR primers used in this study. [file 11658_2023_440_MOESM3_ESM.pdf]

Supplementary Table S3.

PCR primers used in this study.

Primer	Forward	Reverse
POL-RT1	CAG CCT ACT CCC ATC TCT CCA CCT CTA AG-3	GCT CCA GAC CGG CTG CGA GC-3
POL-RT2	CCT CAG GCC ATG CAG TGG AA	GTA TGG ATC GGC AGA GGA GC
rt269I-C	GAA CAT ATT GTA CAA AAA ATC AAG CAA TGT TTT CGG AAA	TTT CCG AAA ACA TTG CTT GAT TTT TTG TAC AAT ATG TTC
Human mitochondrial DNA 1	CATGCCCATCGTCCTAGAAT	ACGGGCCCTATTTCAAAGAT
Human mitochondrial DNA 2	CCCTAACACCAGCCTAACCA	AA AGTGCATACCGCC7AAAAG
Human mitochondrial DNA 3	TCCAACT CATGAGACCCACA	TGAGGCT TGGATTAGCGTTT
Human ATF4	GAC CGA AAT GAG CTT CCT GA	ACC CAT GAG GTT TGA AGT GC
Human ATF4	TCT CAT TCA GGC TTC TCA CGG CAT	AAG CTC ATT TCG GTC ATG TTG CGG
Human ATF6 EMDM1	TTC CCT CCT GGT GGA ATT TG	AGG CCA CTC TGC TTT CCA AC
Human XBP1s	TGC TGA GTC CGC AGC AGG TG	GCT GGC AGG CTC TGG GGA AG
Human Beclin 1	ACC GTG TCA CCA TCC AGG AA	GAA GCT GTT GGC ACT TTC TGT
Human LC3	GAG AAG CAG CTT CCT GTT CTG G	GTG TCC GTT CAC CAA CAG GAA G
Human NADH dehydrogenase	ATA CCC ATG GCC AAC CTC CT	GGG CCT TTG CGT AGT TGT AT
Human cytochrome c oxidase	ATG ACC CAC CAA TCA CAT GC	ATC ACA TGG CTA GGC CGG AG

β-Actin	ATT GCC GAC AGG ATG CAG AA	GCT GAT CCA CAT CTG CTG GAA
