# Supplementary material for: rt269L-Type hepatitis B virus (HBV) in genotype C infection leads to improved mitochondrial dynamics via the PERK–eIF2α–ATF4 axis in an HBx protein-dependent manner
Source: Cell Mol Biol Lett. 2023 Mar 30;28:26. doi: 10.1186/s11658-023-00440-1 (PMC10064691; doi:10.1186/s11658-023-00440-1)
Supplement: Supplementary file 4 — Additional file 4: Table S4. List of Antibodies. [file 11658_2023_440_MOESM4_ESM.pdf]

Supplementary Table S4. List of Antibodies.

Antibody Name	Company
Anti- GAPDH	Santa Cruz Biotechnology (Dallas, TX, USA), sc-25778 and sc-293335
Anti- PGC1α	Santa Cruz Biotechnology (Dallas, TX, USA), sc-517380
Anti-PreS1(AP1)	Santa Cruz Biotechnology (Dallas, TX, USA), sc-57761
Anti- PERK	Cell Signaling Technology (Danvers, MA, USA), #3192
Anti- phospho-PERK	Cell Signaling Technology (Danvers, MA, USA), #3179<br>Invitrogen (Carlsbad, CA, USA), PA5-102853
Anti- ATF4	Cell Signaling Technology (Danvers, MA, USA), #11815
Anti-phospho-eIF2α	Cell Signaling Technology (Danvers, MA, USA), #3597
Anti- IRE1α	Cell Signaling Technology (Danvers, MA, USA), #3294
Anti- cleaved-caspase 3	Cell Signaling Technology (Danvers, MA, USA), #9661
anti- LC3B	Cell Signaling Technology (Danvers, MA, USA), #2775
Anti-CHOP	Cell Signaling Technology (Danvers, MA, USA), #2895
Anti-PI3KC3	Cell Signaling Technology (Danvers, MA, USA), #4263
Anti-PI3K	Cell Signaling Technology (Danvers, MA, USA), #4228
Anti-phosphoAkt	Cell Signaling Technology (Danvers, MA, USA), #9271
Anti-ATF6	Proteintech Group, Inc (Rosemont, IL, USA), 24169-1-AP
Anti-Beclin 1	Abcam (Cambridge, UK), ab62557
Anti-mTOR (phospho)	Abcam (Cambridge, UK), ab109268
Anti-PINK1	Abcam (Cambridge, UK), ab23707
Anti-Parkin	Abcam (Cambridge, UK), ab77924
Anti-phospho-IRE1α	Abcam (Cambridge, UK), ab48187<br>Invitrogen (Carlsbad, CA, USA), PA1-16927
Anti-Flag M2 antibody	Sigma–Aldrich (St. Louis, MO, USA), F1804
Alexa Fluor 488-conjugated goat anti-rabbit IgG (H+L)	Invitrogen (Carlsbad, CA, USA), A-11008
Alexa Fluor 594-conjugated goat	Invitrogen (Carlsbad, CA, USA), A-11005

anti-mouse IgG (H+L)	
MitoSOX Red mitochondrial superoxide indicator	Invitrogen (Carlsbad, CA, USA), M36008
MitoTracker, Deep Red FM	Invitrogen (Carlsbad, CA, USA), M22426,
MitoTracker, Green FM	Invitrogen (Carlsbad, CA, USA), M7514
Tunel apoptosis detection kit (DNA fragmentation/fluorescence staining)	Millipore, (Billerica, MA, USA), 17-141
Bafilomycin-A1	Sigma–Aldrich (St. Louis, MO, USA), B 1793
