# Supplementary material for: rt269L-Type hepatitis B virus (HBV) in genotype C infection leads to improved mitochondrial dynamics via the PERK–eIF2α–ATF4 axis in an HBx protein-dependent manner
Source: Cell Mol Biol Lett. 2023 Mar 30;28:26. doi: 10.1186/s11658-023-00440-1 (PMC10064691; doi:10.1186/s11658-023-00440-1)
Supplement: Supplementary file 5 — Additional file 5: Figure S1. Related to Fig. 1., rt269L type showed improved mitochondrial maintenance, with high rate of functional mitochondria and biogenesis. Confocal microscopy images showing HepG2 cells transfected with mock, rt269L, or rt269I HBV followed by TMRM staining. Mitochondrial outer membrane permeabilization (MOMP) and ΔΨm were observed. Nuclei were stained with DAPI (blue). Scale bar, 50 μm [file 11658_2023_440_MOESM5_ESM.pdf]

Figure S1.

DAPI/TMRM

Mock	rt269L	rt269I

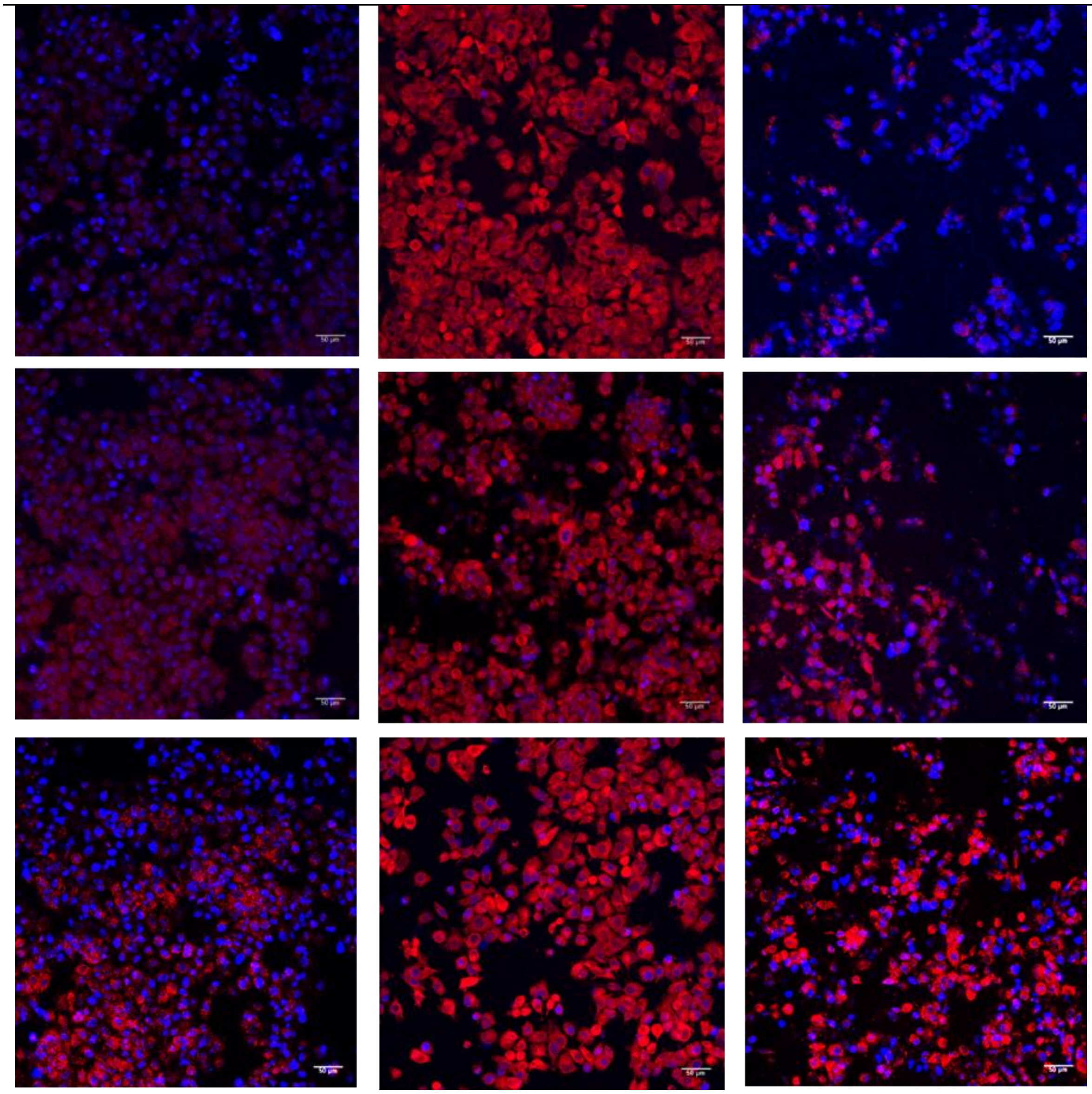

Fig. S1., related to Fig. 1., rt269L type showed improved mitochondrial maintenance with high rate of functional mitochondria and biogenesis Confocal microscopy images showing HepG2 cells transfected with mock, rt269L, or rt269I HBV followed by TMRM staining. Mitochondrial outer membrane permeabilization (MOMP) and ΔΨm were observed. Nuclei were stained with DAPI (blue). Scale bar = 50 µm.
