# Supplementary material for: rt269L-Type hepatitis B virus (HBV) in genotype C infection leads to improved mitochondrial dynamics via the PERK–eIF2α–ATF4 axis in an HBx protein-dependent manner
Source: Cell Mol Biol Lett. 2023 Mar 30;28:26. doi: 10.1186/s11658-023-00440-1 (PMC10064691; doi:10.1186/s11658-023-00440-1)
Supplement: Supplementary file 6 — Additional file 6: Figure S2. A WT (rt269L) HBV induced higher transcription of three different mtDNA genes than in rt269I HBV infection. Mitochondrial copy numbers were calculated based on RT–qPCR results obtained with three different mtDNA primer sets. The results were evaluated for statistical significance by one-way ANOVA with Tukey’s post hoc test or t-test. Differences were considered significant when *p < 0.05, **p < 0.01, or ***p < 0.001. B. Protein expression levels were analyzed by western blotting with antibodies against PGC1α and GAPDH. The relative intensity compared with GAPDH was analyzed. *p < 0.05, **p < 0.01, ***p < 0.001 [file 11658_2023_440_MOESM6_ESM.pdf]

Figure S2.

(A)

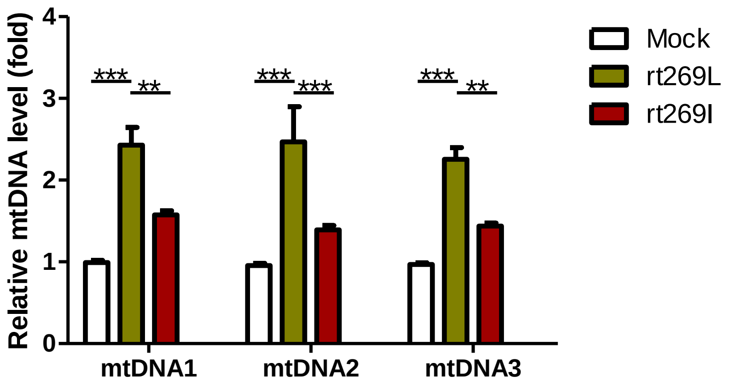

(B)

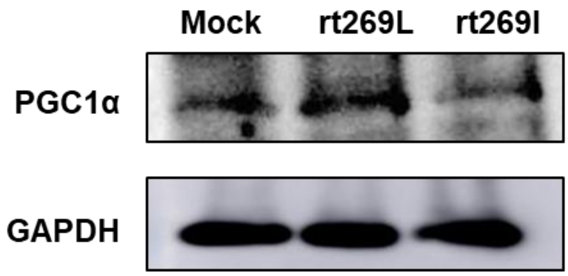

Fig. S2A.WT (rt269L) HBV induced higher transcription of three different mtDNA genes than rt269I HBV infection Mitochondrial copy numbers were calculated based on RT–qPCR results obtained with three different mtDNA primer sets. The results were evaluated for statistical significance by one-way ANOVA with Tukey's post-hoc test or t-test. Differences were considered significant when $*p < 0.05$, $**p < 0.01$, or $***p < 0.001$.

Fig. S2B. Protein expression levels were analyzed by Western blotting with antibodies against PGC1α and GAPDH. The relative intensity compared to GAPDH was analyzed. $*p < 0.05$, $**p < 0.01$, $***p < 0.001$.
