# Supplementary material for: rt269L-Type hepatitis B virus (HBV) in genotype C infection leads to improved mitochondrial dynamics via the PERK–eIF2α–ATF4 axis in an HBx protein-dependent manner
Source: Cell Mol Biol Lett. 2023 Mar 30;28:26. doi: 10.1186/s11658-023-00440-1 (PMC10064691; doi:10.1186/s11658-023-00440-1)
Supplement: Supplementary file 7 — Additional file 7: Figure S3. WT (rt269L) HBV induces ER stress and activates the PERK-mediated pathway in the unfolded protein response (UPR). Immunohistochemical analysis of p-eIF2α expression in paraffin-embedded liver tissues (magnification 100×, n = 5 per group) [file 11658_2023_440_MOESM7_ESM.pdf]

Figure S3.

Mock	rt269L	rt269I

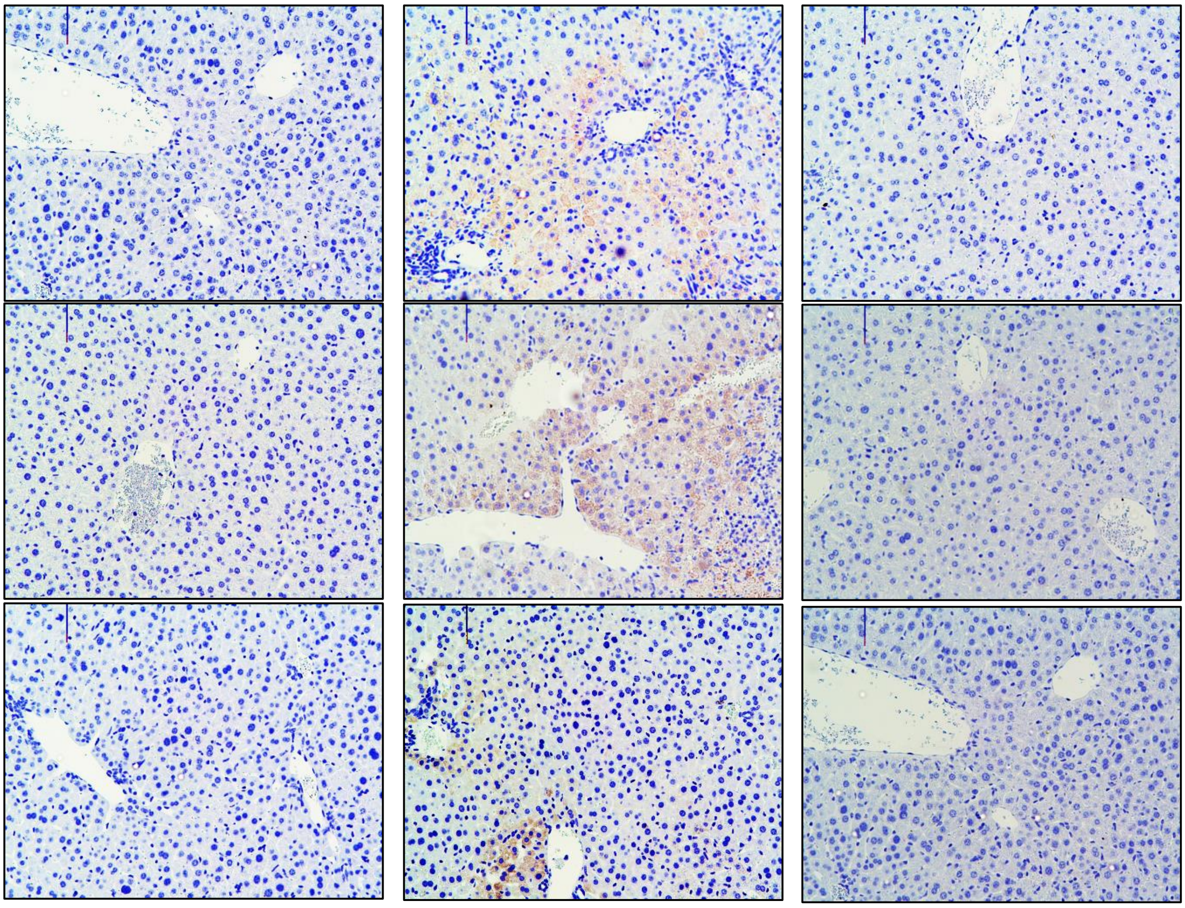

Fig. S3. WT (rt269L) HBV induces ER stress and activates the PERK-mediated pathway in the unfolded protein response (UPR) Immunohistochemical analysis of p-eIF2α expression in paraffin-embedded liver tissues (magnification 100x, n=5 per group).
