# Supplementary material for: rt269L-Type hepatitis B virus (HBV) in genotype C infection leads to improved mitochondrial dynamics via the PERK–eIF2α–ATF4 axis in an HBx protein-dependent manner
Source: Cell Mol Biol Lett. 2023 Mar 30;28:26. doi: 10.1186/s11658-023-00440-1 (PMC10064691; doi:10.1186/s11658-023-00440-1)
Supplement: Supplementary file 8 — Additional file 8: Figure S4. EGFP-LC3 assay with flow cytometry. EGFP-LC3B-positive HepG2 cells cotransfected with mock, rt269L, or rt269I HBV were detected by flow cytometry [file 11658_2023_440_MOESM8_ESM.pdf]

Figure S4.

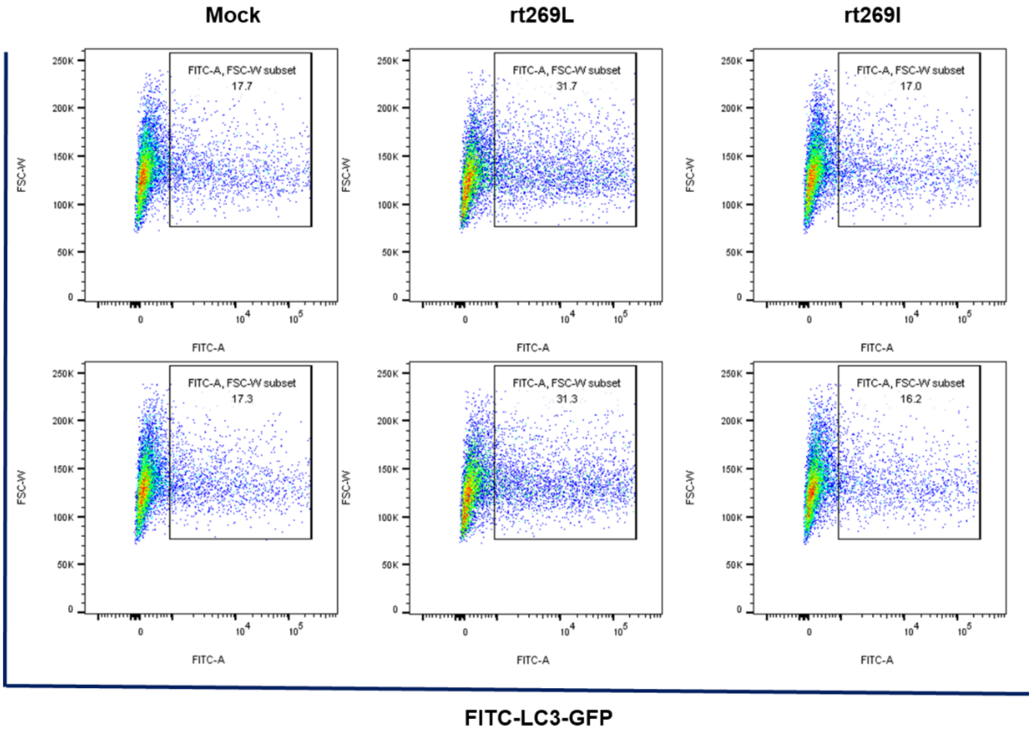

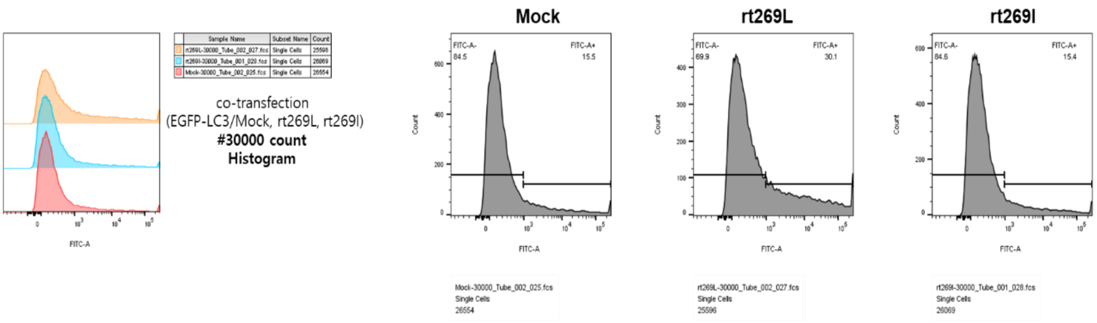

Fig. S4. EGFP-LC3 assay with flow cytometry. EGFP-LC3B-positive HepG2 cells cotransfected with mock, rt269L, or rt269I HBV were detected by flow cytometry.
