# Supplementary material for: rt269L-Type hepatitis B virus (HBV) in genotype C infection leads to improved mitochondrial dynamics via the PERK–eIF2α–ATF4 axis in an HBx protein-dependent manner
Source: Cell Mol Biol Lett. 2023 Mar 30;28:26. doi: 10.1186/s11658-023-00440-1 (PMC10064691; doi:10.1186/s11658-023-00440-1)
Supplement: Supplementary file 9 — Additional file 9: Figure S5. A Impaired autophagy in rt269I HBV infection western blots showing the autophagy marker protein LC3. B HBx in rt269L strongly interact with PI3KC3 (VPS34). HBx-flag plasmid was cotransfected with mock, genoC-L, genoC-I, genoA-L, or genoC-I plasmid in HepG2 cells, as indicated. At 42 h posttransfection, cells were extracted and the cell lysates were subjected to IP with anti-Flag Ab followed by IB using anti-PI3KC3 Ab [file 11658_2023_440_MOESM9_ESM.pdf]

Figure S5A

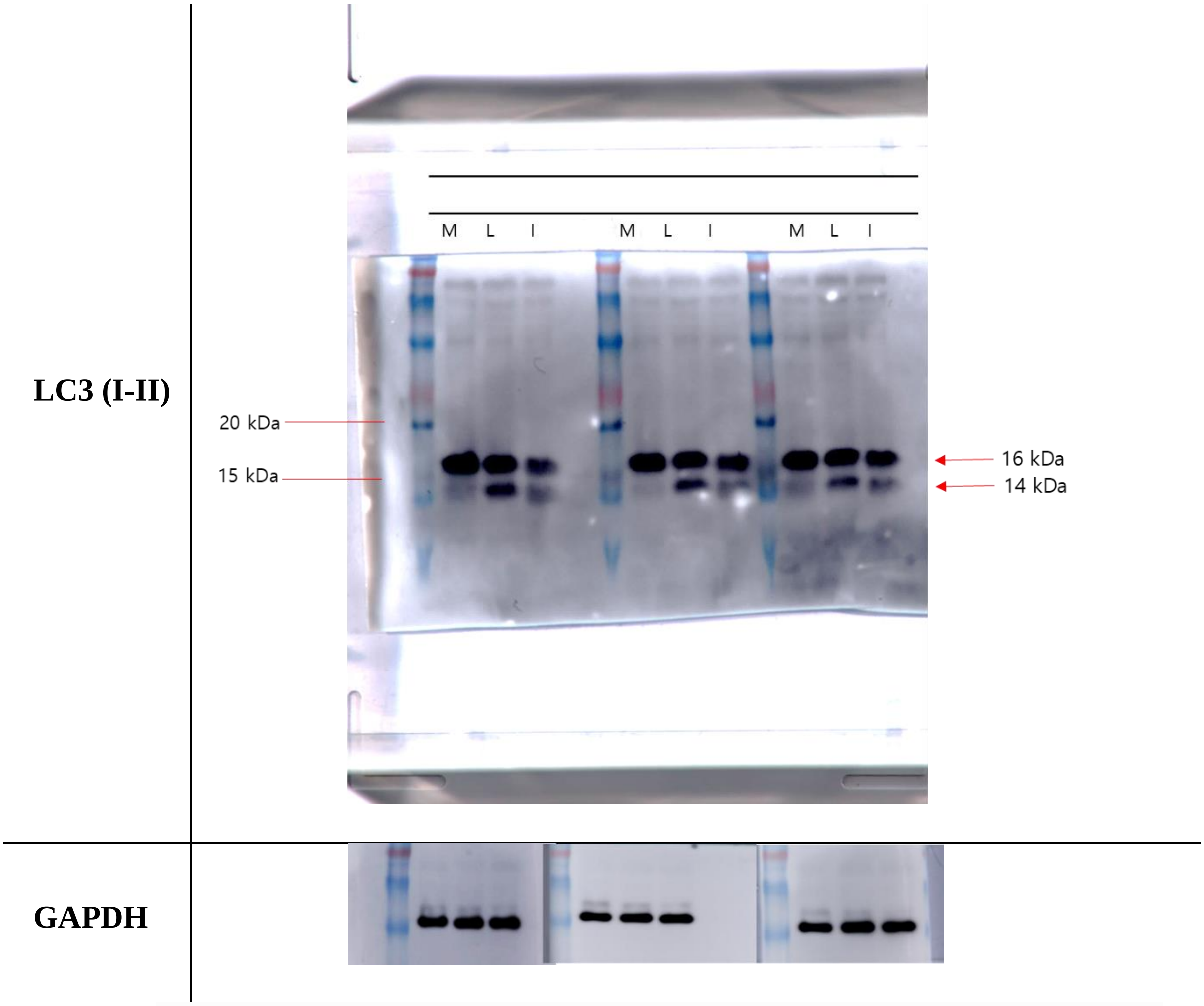

Fig. S5A. Impaired autophagy in rt269I HBV infection Western blots showing the autophagy marker protein LC3.

Figure S5B

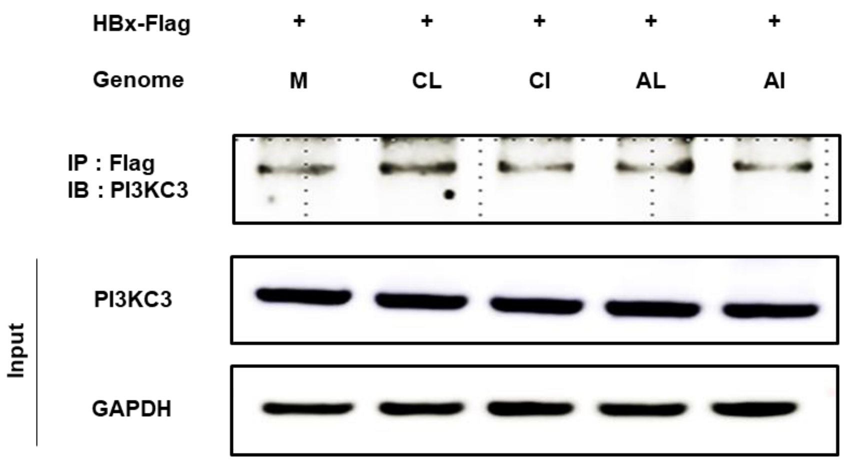

Fig. S5B HBx in rt269L strongly interact with PI3KC3 (VPS34) HBx-flag plasmid was cotransfected with Mock, GenoC-L, genoC-I, genoA-L, or genoC-I plasmid in HepG2 cells, as indicated. At 42 h post-transfection, cells were extracted and the cell lysates were subjected to IP with anti-Flag Ab followed by IB using anti-PI3KC3 Ab.
