# Supplementary material for: rt269L-Type hepatitis B virus (HBV) in genotype C infection leads to improved mitochondrial dynamics via the PERK–eIF2α–ATF4 axis in an HBx protein-dependent manner
Source: Cell Mol Biol Lett. 2023 Mar 30;28:26. doi: 10.1186/s11658-023-00440-1 (PMC10064691; doi:10.1186/s11658-023-00440-1)
Supplement: Supplementary file 10 — Additional file 10: Figure S6. Immunofluorescence for the colocalization of autophagosomes with mitochondria. EGFP-LC3-expressing vector was cotransfected with HBV genome plasmids. Mitochondria were stained with MitoTracker deep red, and yellow dots represent colocalized EGFP-LC3-positive autophagosomes with red labeled mitochondria [file 11658_2023_440_MOESM10_ESM.pdf]

Figure S6

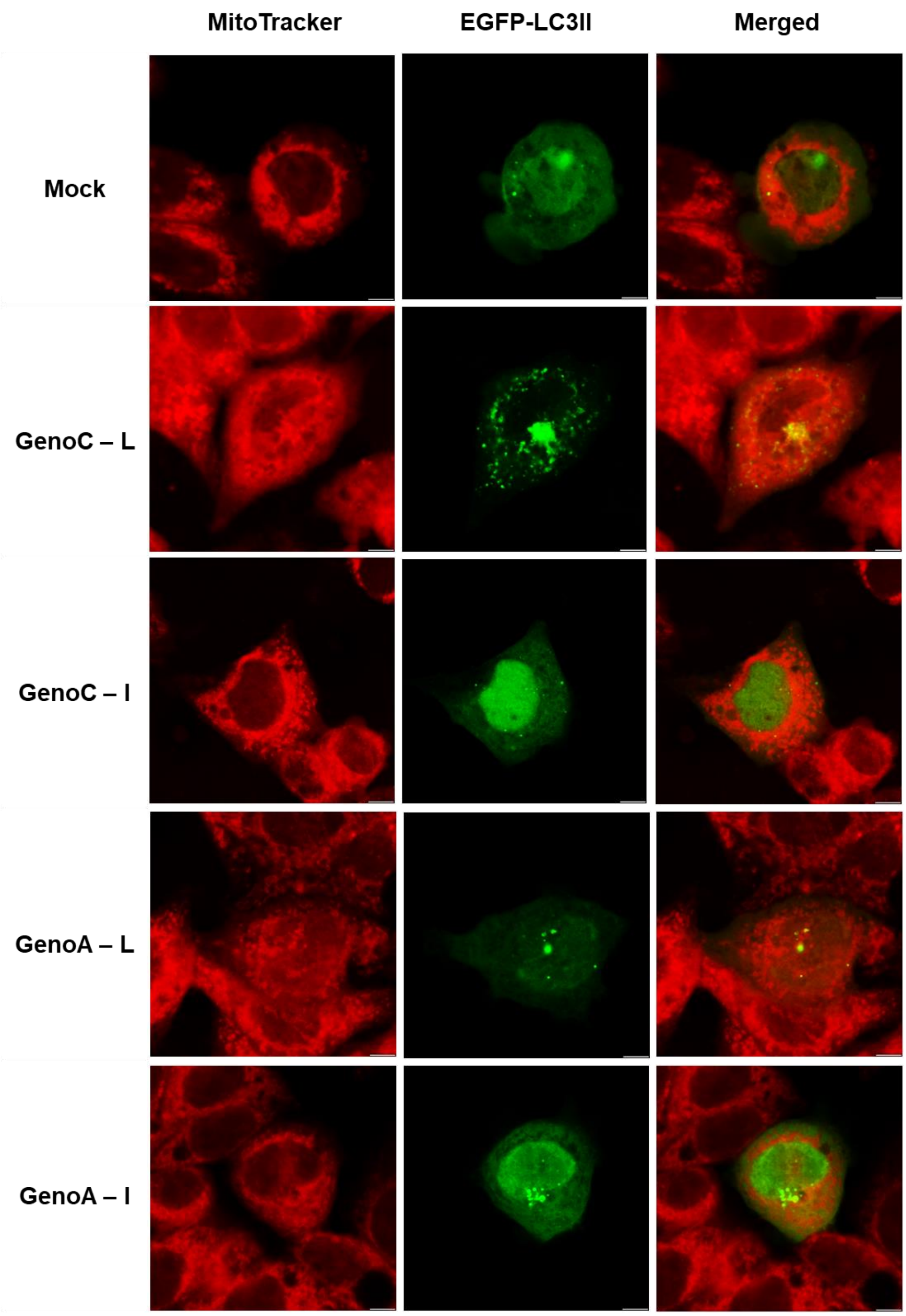

Fig.S6. Immunofluorescence for the colocalization of autophagosomes with mitochondria. EGFP-LC3-expressing vector was cotransfected with HBV genome plasmids. Mitochondria were stained with MitoTracker deep red and yellow dots represent colocalized EGFP-LC3-positive autophagosomes with red labeled mitochondria.
