# Supplementary material for: rt269L-Type hepatitis B virus (HBV) in genotype C infection leads to improved mitochondrial dynamics via the PERK–eIF2α–ATF4 axis in an HBx protein-dependent manner
Source: Cell Mol Biol Lett. 2023 Mar 30;28:26. doi: 10.1186/s11658-023-00440-1 (PMC10064691; doi:10.1186/s11658-023-00440-1)
Supplement: Supplementary file 11 — Additional file 11: Figure S7. rt269I HBV induces caspase activation and cell death Immunohistochemistry analysis of cleaved caspase-3 expression in paraffin-embedded liver tissues (magnification 100×, n = 5 per group) [file 11658_2023_440_MOESM11_ESM.pdf]

Figure S7

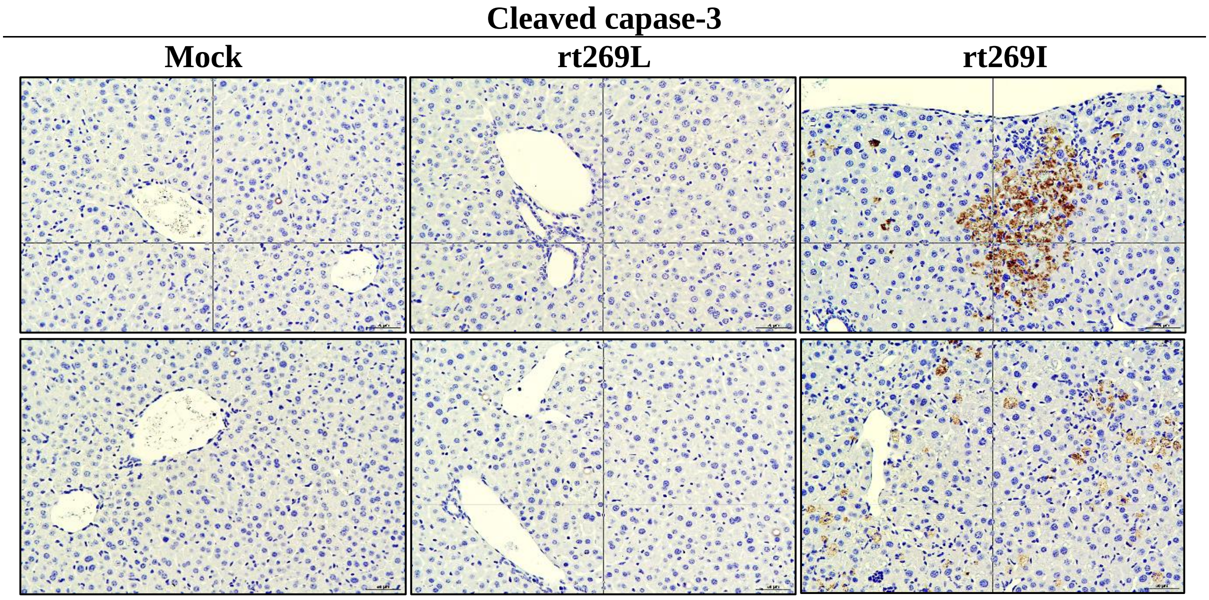

Fig. S7. rt269I HBV induces caspase activation and cell death Immunohistochemistry analysis of cleaved caspase-3 expression in paraffin-embedded liver tissues (magnification 100x, n=5 per group).
