# Supplementary material for: rt269L-Type hepatitis B virus (HBV) in genotype C infection leads to improved mitochondrial dynamics via the PERK–eIF2α–ATF4 axis in an HBx protein-dependent manner
Source: Cell Mol Biol Lett. 2023 Mar 30;28:26. doi: 10.1186/s11658-023-00440-1 (PMC10064691; doi:10.1186/s11658-023-00440-1)
Supplement: Supplementary file 12 — Additional file 12. Figure S8. Detection of intracellular reactive oxygen species in hepatocytes transfected with rt269L or rt269I HBV. Reactive oxygen species (ROS) were detected with a CM-H2DCFDA fluorescence probe in hepatocytes transfected with the rt269L or rt269I HBV vector. H2O2 was used as the positive control for ROS induction [file 11658_2023_440_MOESM12_ESM.pdf]

Figure S8

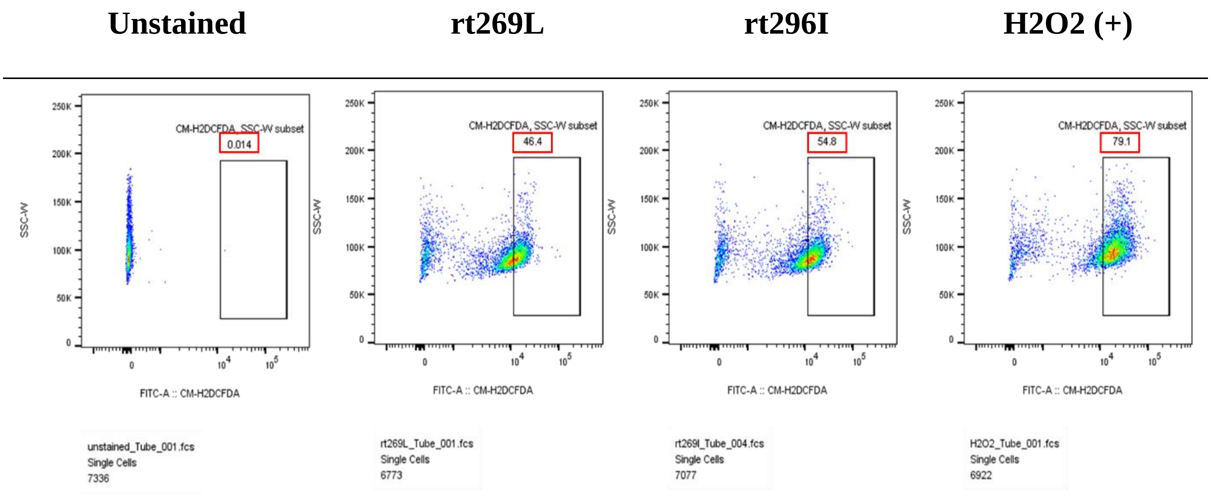

Fig. S8. Detection of intracellular reactive oxygen species in hepatocytes transfected with rt269L or rt269I HBV Reactive oxygen species (ROS) were detected with a CM-H2DCFDA fluorescence probe in hepatocytes transfected with the rt269L or rt269I HBV vector. H_2O_2 was used as the positive control for ROS induction.
