# Supplementary material for: rt269L-Type hepatitis B virus (HBV) in genotype C infection leads to improved mitochondrial dynamics via the PERK–eIF2α–ATF4 axis in an HBx protein-dependent manner
Source: Cell Mol Biol Lett. 2023 Mar 30;28:26. doi: 10.1186/s11658-023-00440-1 (PMC10064691; doi:10.1186/s11658-023-00440-1)
Supplement: Supplementary file 13 — Additional file 13: Figure S9. Cell death in liver tissues of mice hydrodynamically injected with the mock, rt269L, or rt269I vector was detected by TUNEL assay (FITC-conjugated). Nuclei were stained with DAPI (blue) [file 11658_2023_440_MOESM13_ESM.pdf]

Figure S9

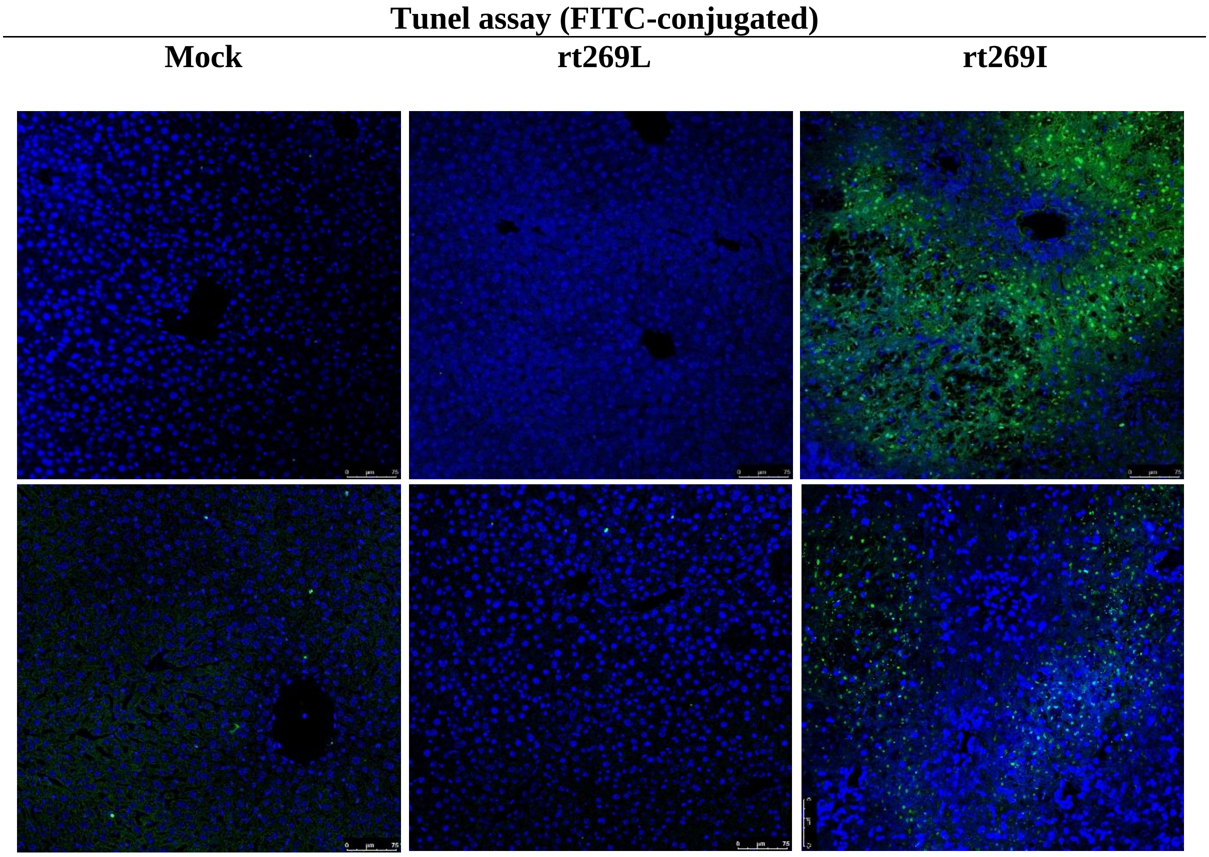

Fig. S9. Cell death in liver tissues of mice hydrodynamically injected with the mock, rt269L, or rt269I vector was detected by TUNEL assay (FITC-conjugated). Nuclei were stained with DAPI (blue).
