# Supplementary material for: rt269L-Type hepatitis B virus (HBV) in genotype C infection leads to improved mitochondrial dynamics via the PERK–eIF2α–ATF4 axis in an HBx protein-dependent manner
Source: Cell Mol Biol Lett. 2023 Mar 30;28:26. doi: 10.1186/s11658-023-00440-1 (PMC10064691; doi:10.1186/s11658-023-00440-1)
Supplement: Supplementary file 14 — Additional file 14: Figure S10. rt269L-type HBV infection, in both genotype C and A, activated phospho-Akt and phospho-PI3K signals. Western blot analysis of the phospho-PI3K and phosphor-Akt, and GAPDH. The relative intensity was analyzed. **p < 0.01, ***p < 0.001 [file 11658_2023_440_MOESM14_ESM.pdf]

Figure S10

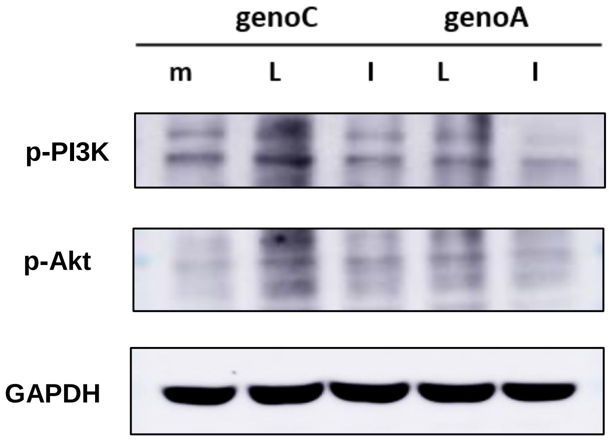

Fig. S10. rt269L type HBV infection in both genotype C and A activated phospho-Akt and phospho-PI3K signals Western blot analysis of the phospho-PI3K and phosphor-Akt, and GAPDH. The relative intensity was analyzed. $^{**}p < 0.01$, $^{***}p < 0.001$.
