# Supplementary material for: rt269L-Type hepatitis B virus (HBV) in genotype C infection leads to improved mitochondrial dynamics via the PERK–eIF2α–ATF4 axis in an HBx protein-dependent manner
Source: Cell Mol Biol Lett. 2023 Mar 30;28:26. doi: 10.1186/s11658-023-00440-1 (PMC10064691; doi:10.1186/s11658-023-00440-1)
Supplement: Supplementary file 15 — Additional file 15: Figure S11. rt269L-type HBV infection activated phospho-mTOR and phospho-PI3K signals in HBx protein dependent manner. Western blot analysis of the phospho-PI3K and phospho-mTOR, and GAPDH. The relative intensity was analyzed. **p < 0.01, ***p < 0.001 [file 11658_2023_440_MOESM15_ESM.pdf]

Figure S11

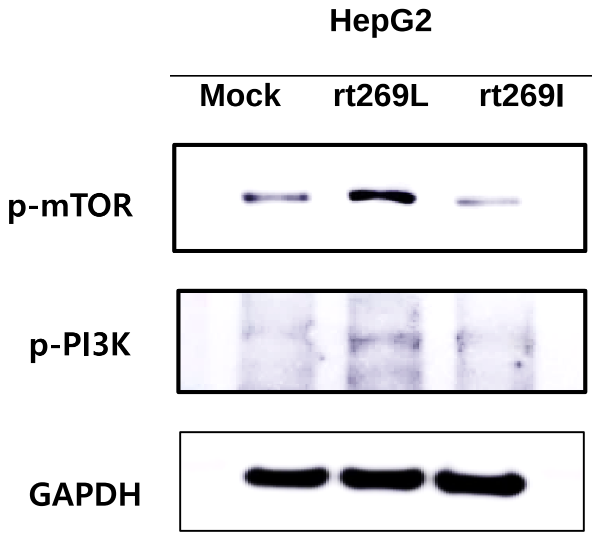

Fig. S11. rt269L type HBV infection activated phospho-mTOR and phospho-PI3K signals in HBx protein dependent manner Western blot analysis of the phospho-PI3K and phospho-mTOR, and GAPDH. The relative intensity was analyzed. **$p < 0.01$, ***$p < 0.001$.
