# Supplementary material for: rt269L-Type hepatitis B virus (HBV) in genotype C infection leads to improved mitochondrial dynamics via the PERK–eIF2α–ATF4 axis in an HBx protein-dependent manner
Source: Cell Mol Biol Lett. 2023 Mar 30;28:26. doi: 10.1186/s11658-023-00440-1 (PMC10064691; doi:10.1186/s11658-023-00440-1)
Supplement: Supplementary file 16 — Additional file 16: Figure S12. Site-directed mutagenesis to introduce a stop codon downstream of the polymerase start region [file 11658_2023_440_MOESM16_ESM.pdf]

Figure S12

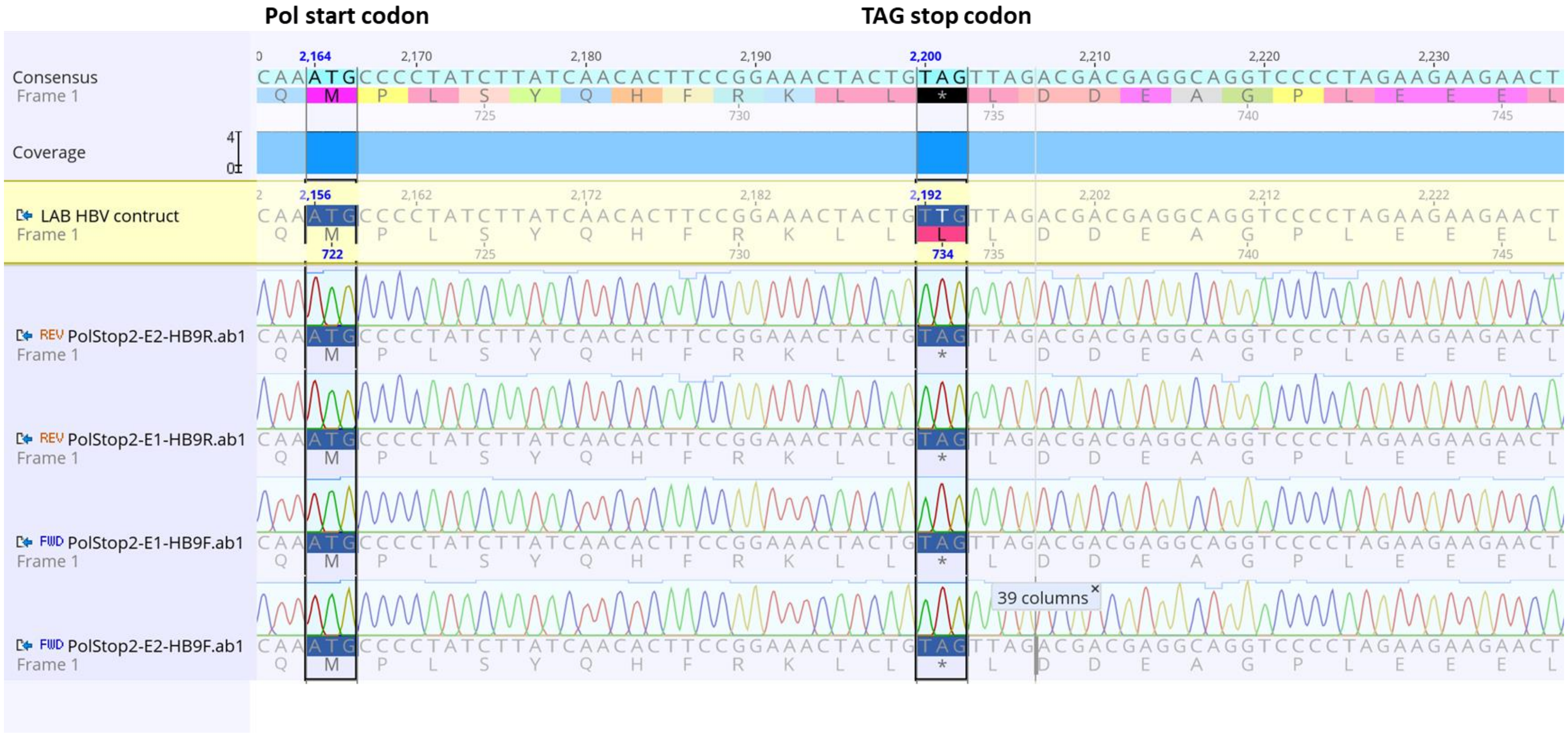

Fig. S12 Site-directed mutagenesis to introduce a stop codon downstream of the Polymerase start region

A stop codon was inserted at 36 bp downstream of Polymerase start region (Met) by site-directed point mutation. Converted from TTG to TAG (stop), prevented the translation of full-length Pol region.
