# Supplementary material for: rt269L-Type hepatitis B virus (HBV) in genotype C infection leads to improved mitochondrial dynamics via the PERK–eIF2α–ATF4 axis in an HBx protein-dependent manner
Source: Cell Mol Biol Lett. 2023 Mar 30;28:26. doi: 10.1186/s11658-023-00440-1 (PMC10064691; doi:10.1186/s11658-023-00440-1)
Supplement: Supplementary file 17 — Additional file 17. Figure S13. Site-directed mutagenesis to introduce a stop codon upstream of the rt269 region. A stop codon was inserted at 49 bp (887 bp) upstream of rt269 region (936 bp) by site-directed point mutation. Conversion from TAT to TAA (stop) prevented the translation of full-length Pol-RT269 region [file 11658_2023_440_MOESM17_ESM.pdf]

Figure S13

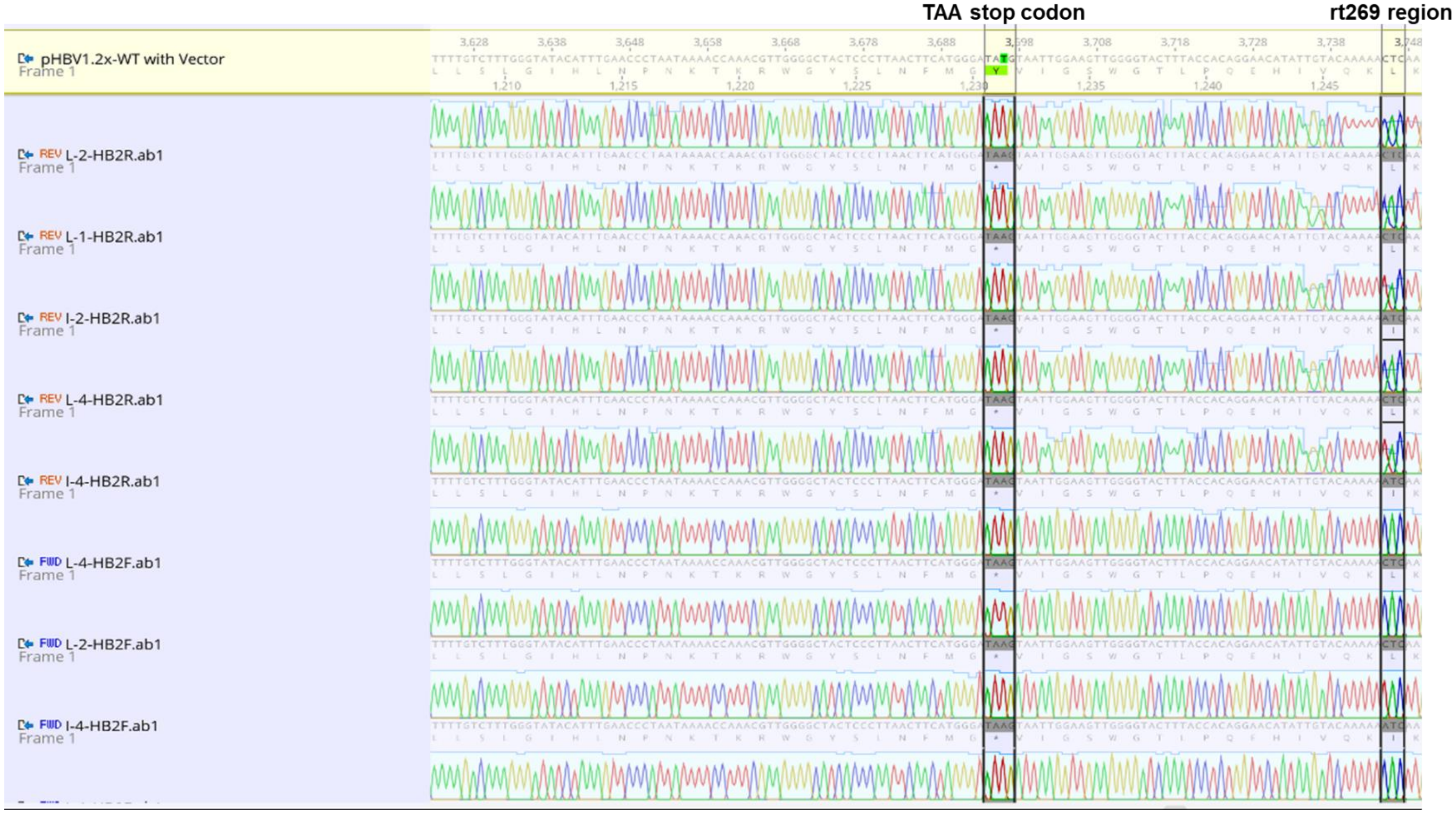

Fig. S13 Site-directed mutagenesis to introduce a stop codon upstream of the rt269 region

A stop codon was inserted at 49 bp (887 bp) upstream of rt269 region (936 bp) by site-directed point mutation. Converted from TAT to TAA (stop), prevented the translation of full-length Pol-RT269 region.
