# Supplementary material for: rt269L-Type hepatitis B virus (HBV) in genotype C infection leads to improved mitochondrial dynamics via the PERK–eIF2α–ATF4 axis in an HBx protein-dependent manner
Source: Cell Mol Biol Lett. 2023 Mar 30;28:26. doi: 10.1186/s11658-023-00440-1 (PMC10064691; doi:10.1186/s11658-023-00440-1)
Supplement: Supplementary file 18 — Additional file 18. Materials and methods [file 11658_2023_440_MOESM18_ESM.pdf]

rt269L infection in HBV genotype C leads to improved mitochondrial dynamics via the PERK-eIF2α-ATF4 axis in an HBx protein-dependent manner

Yu-Min Choi, Dong Hyun Kim, Junghwa Jang, Won Hyeok Choe, and Bum-Joon Kim

Supporting Materials

Supplementary Materials and Methods

Supplementary Materials and Methods

HBV DNA extraction and PCR amplification of the polymerase RT region

For this cohort study, HBV DNA was extracted from the serum of patients using a QIAamp DNA Blood Mini kit (QIAGEN, Hilden, Germany), and the sample was dissolved in Tris-EDTA buffer (10 mM Tris-HCl and 1 mM ethylenediaminetetraacetic acid, pH 8.0). First-round PCR was performed using primers POL-RT1 and the amplicon was used as a template for second-round PCR using primers POL-RT2 (Table S1). The PCR products were subjected to direct sequencing analysis.

HBV genotyping

A total of 1,032-bp polymerase RT sequences were examined by direct sequencing and compared to the sequences of the reference strains representing each of the genotype (A-H including the C strains) obtained from GenBank. The sequences of the RT region were compared via the Bayesian method in the phylogenetic/molecular evolutionary analysis with MrBayes version 3.2.7, and the phylogenetic tree was constructed using FigTree version 1.4.3. The maximum-likelihood method was also used for the phylogenetic analysis with MEGA version 10.0. Phylogenetic trees were reconstructed using 1,000 bootstrap replicates, and the mean genetic distances were estimated using the Kimura two-parameter with invariant sites and gamma model.

Plasmid and site-directed mutagenesis

pHBV-1.2x (GenBank accession No. AY641558) containing the full-length HBV genotype C genome was used for site-directed mutagenesis to generate polymerase RT mutant DNA constructs with an *i*-pfu kit (iNtRON, Seongnam, Korea). Mutagenesis was

performed using the primer rt269I based on the rt269L construct. To exclude the CMV promoter, HBV full-genome constructs were cut by the restriction enzyme *Sma*I and prepared for linear genome formation.

Transfection

HepG2 human hepatocellular carcinoma cells and Huh7 cells purchased from the Korean Cell Line Bank (KCLB, Seoul, South Korea) were grown at 37°C in a humidified environment containing 5% CO_2. pHBV-1.2x containing the full-length HBV genotype C genome (2.5 μg) was transiently transfected using Lipofectamine 3000 (Invitrogen, Carlsbad, CA, USA). To normalize the transfection efficacy data, pSV-β-galactosidase (0.25 μg) was cotransfected, and the enzyme assay was performed using a β-Galactosidase Enzyme Assay System with Reporter Lysis buffer (Promega, Madison, WI, USA) following the manufacturer’s protocol.

Total RNA extraction and real-time polymerase chain reaction (RT–qPCR)

Total RNA was extracted from transfected cells or mouse liver tissue using TRIzol, and the target genes were amplified with SensiFAST SYBR Lo-ROX One-Step kits (BioLine, London, UK). The transcription level was analyzed using qRT–PCR with the primer sets shown in Table S1, and the housekeeping gene β-actin was used as the internal control.

Preparation of HBV from transiently transfected cells and infection assay

For the infection experiments, the supernatant of the culture medium of HepG2 cells transiently transfected with 1.2x-rt269L or rt269I HBV plasmids was collected. The supernatant was purified through a sterile 0.45-μm pore size filter and precipitated with 6% polyethylene glycol (PEG) 8000 overnight. The medium was ultracentrifuged, and the collected pellet was resuspended in PBS containing 15–25% fetal calf serum (FCS). After quantification by qPCR, 3×10^9 HBV genome equivalents per milliliter were aliquoted and stored at −80°C. HepG2-hNTCP-C4 cells were seeded in 6-well plates. The infection assay was performed with concentrated virus in the presence of 4% PEG8000 at 37°C for 20 h. The HepG2-hNTCP-C4 cells were kindly provided by Dr. Koichi Watashi (National Institute of Infectious Disease, Tokyo, Japan).

Immunofluorescence analysis

Cells were seeded and cultivated in two-chamber glass slides (Nunc, Roskilde, Denmark) for 12 h before each experiment. The cells were fixed with 4% paraformaldehyde (PFA) and permeabilized with 0.25% Triton-X 100 for 10 min. The cells were stained with primary antibodies (1:100, overnight at 4°C) and secondary antibodies (1:1000, 2 h at room temperature) in 1% bovine serum albumin (BSA) in PBST and mounted in mounting medium containing DAPI (VECTASHIELD, Vetor Laboratories, Inc., Burlingame, CA, USA). Images were captured and analyzed using software to quantify the staining intensity (Leica Software analysis, LAS X and ImageJ program, version 1.52a).

Western blot analysis

The harvested cells were lysed with RIPA buffer (CST, #9806) containing protease and

phosphatase inhibitors (Hoffmann-La Roche Inc., Basel, Switzerland) and incubated for 20 min on ice. The lysed cells were centrifuged for 30 min at 13,000 rpm, and the lysates were collected for Western blotting. Protein samples were separated by electrophoresis, transferred to nitrocellulose membranes, and blocked for 1 h with 5% skim milk or BSA. The membranes were incubated overnight at 4°C with the primary antibodies (1:1000). The next day, the membranes were washed with 0.1% Tween-20 in Tris-buffered saline and incubated with horseradish peroxidase-conjugated secondary antibodies (1:10000) for 2 h. After the ECL solution was applied to the membrane, proteins were detected on an imager (AI680).

Mitochondrial membrane potential (ΔΨm) assay

JC-1 staining

To determine the ΔΨm, the dual emission potentiometric dye JC-1 was used. JC-1 aggregates fluoresce red (～597 nm), whereas monomers fluoresce green (～539 nm). The relative intensity of red and green fluorescence was used to indicate the variation in ΔΨm. Cells were incubated with 1 μM JC-1 (MCE, Monmouth Junction NJ, USA). Images were acquired with an Olympus FV3000 confocal microscope (Olympus, Tokyo, Japan).

Tetramethylrhodamine methyl ester (TMRM) staining

Cells were incubated in medium containing 250 nM TMRM (Invitrogen) for 30 min at 37°C and 5% CO_2 in the dark. After staining, the cells were washed three times with PBS and mounted in mounting medium containing DAPI (VECTASHIELD, Vetor Laboratories, Inc., Burlingame, CA, USA). Images were captured using an Olympus FV3000 confocal microscope (Olympus, Tokyo, Japan).

Mitochondrial functionality assay

A 48-well culture plate was seeded with 1 $\times 10^{6}$ cells. After washing with PBS, the cells were gently scraped from the plate with a cell scraper. The cells were added to a 96-well round-bottom plate. The cells were resuspended in a prewarmed antibody and probe mixture (MitoTracker Red and Green) and incubated for 15 min at 37°C. After staining, the cells were resuspended in PBS + 2% FCS for analysis by flow cytometry. Functional mitochondria fluoresce MitoTracker Green^{high} and MitoTracker Red^{high}, while dysfunctional mitochondria fluoresce MitoTracker Green^{high} and MitoTracker Red^{low} (1).

Immunohistochemistry (IHC)

The liver sections were fixed with 4% paraformaldehyde for 72 h in 4°C and embedded in paraffin. The embedded tissues were cut into 4 ~ 6-μm-thick sections and deparaffinized with a xylene/ethanol solution. Antigen retrieval was performed with the heat-mediated method (sodium citrate buffer), and endogenous peroxidase was blocked by 3% H2O2. For IHC staining, the sections were incubated with primary and secondary antibodies, and chromogenic detection was developed through the conversion of 3,3′-diaminobenzidine (DAB) to a brownish precipitate that remained permanently detectable on slides and were visualized by light microscopy.

Transmission electron microscopy (TEM)

The cells were fixed overnight in a mixture of cold 2.5% glutaraldehyde in 0.1 M phosphate buffer (pH 7.2), and 2% paraformaldehyde in 0.1 M phosphate (pH 7.2). These tissues were postfixed for 1.5 h in 2% osmium tetroxide in 0.1 M phosphate buffer at room

temperature. The cells were then washed briefly with 0.1 M phosphate buffer, dehydrated through a graded 50, 60, 70, 80, 90, 95, and 100% ethanol (X2) series, infiltrated with a propylene oxide and EPON epoxy resin mixture (Embed 812, Nadic methyl anhydride, poly Bed 812, dodecenylsuccinic anhydride, dimethylaminomethyl phenol; Electron Microscopy Polysciences, (USA), and ultimately embedded with only epoxy resin. The epoxy-resin-mixed samples were loaded into capsules and polymerized at 80°C overnight. In preparation for light microscopy analysis, samples were cut into 1.0 nm sections and stained with 1% toluidine blue for 45 sec on a hot plate at 80°C. Thin sections were cut with an ultramicrotome (RMC MT-XL) and collected on a copper grid. Specifically, areas identified for thin sectioning were cut into 65 nm slices and stained with saturated 4% uranyl acetate and 4% lead citrate before examination with a transmission electron microscope (JEM-1400; Japan) at 80 Kv.

XBP1 splicing assay

An X-box-binding protein 1 (XBP1) fragment was PCR amplified from cDNA isolated from HBV-infected or thapsigargin-treated cells using the specific primers pXBP1F, 5′-GGATGCCTTAGTTACTGAAG-3′, and pXBP1R, 5′-GTCCTTCTGGGTCGACTTCT-3′. The PCR amplicons were identified in a 3% agarose gel and visualized using a Gel Documentation System (Bio–Rad, California, USA).

TUNEL assay

For TUNEL assays, tissue slides and cells were reacted with terminal deoxynucleotidyl transferase (TdT) enzyme and fluorescently labeled with 2′-deoxyuridine 5′-triphosphate (dUTP) at 37 °C for 1 h. The nuclei were stained with DAPI. TUNEL-positive cells were

captured and analyzed using software to quantify the staining intensity (Leica Software analysis, LAS X and ImageJ program, version 1.52a).

8-OHdG ELISA Assay

Genomic DNA was extracted from transfected cells using a QIAamp Blood DNA extraction kit (QIAGEN, Hilden, Germany). For the detection of 8-hydroxy-2′-deoxyguanosine (8-OHdG) activity, a competitive ELISA was performed with an 8-OHdG analysis kit (OxiSelect Oxidative DNA Damage ELISA kit, Cell Biolabs, San Diego, CA, USA) according to the manufacturer's protocol.

Cytochrome c release, ATP production, and DNA fragmentation

Cytochrome c release (MCTC0, R&D Systems, Inc. Minneapolis, MN, USA), ATP production (ab83355, Abcam, Cambridge, UK), and DNA fragmentation (#11774425001, Sigma–Aldrich, MO, USA) were determined according to the manufacturer's manual.
